# Testosterone does not affect lower urinary tract symptoms while improving markers of prostatitis in men with benign prostatic hyperplasia: a randomized clinical trial

**DOI:** 10.1007/s40618-022-01776-9

**Published:** 2022-03-17

**Authors:** G. Rastrelli, S. Cipriani, F. Lotti, I. Cellai, P. Comeglio, S. Filippi, V. Boddi, P. A. Della Camera, R. Santi, L. Boni, G. Nesi, S. Serni, M. Gacci, M. Maggi, L. Vignozzi

**Affiliations:** 1 Department of Experimental Clinical and Biomedical Sciences “Mario Serio”, University of Florence, Viale Pieraccini, 6, 50139 Florence, Italy; 2 Interdepartmental Laboratory of Functional and Cellular Pharmacology of Reproduction, Department of Neuroscience, Psychology, Pharmacology and Child Health, University of Florence, Viale Pieraccini, 6, 50139 Florence, Italy; 3 Urology Unit, Azienda Ospedaliera Universitaria Careggi, Largo Piero Palagi, 1, 50139 Florence, Italy; 4 Pathological Anatomy Unit, Careggi University Hospital, Largo Piero Palagi, 1, 50139 Florence, Italy; 5 Unit of Clinical Epidemiology, IRCCS Policlinico San Martino, Largo Rosanna Benzi, 10, 16132 Genoa, Italy; 6 Department of Health Sciences, University of Florence, Viale Pieraccini, 6, 50139 Florence, Italy; 7 Istituto Nazionale Biostrutture e Biosistemi, Viale delle Medaglie d’Oro, 305, 00136 Rome, Italy

**Keywords:** Metabolic syndrome, Testosterone therapy, Benign prostatic hyperplasia, Hypogonadism, LUTS, Prostatitis-like symptoms

## Abstract

**Purpose:**

Benign Prostatic Hyperplasia (BPH) is a result of prostate inflammation, frequently occurring in metabolic syndrome (MetS). Low testosterone is common in MetS. A randomized clinical trial was designed to evaluate if 24 weeks of testosterone therapy (TTh) in BPH men with MetS and low testosterone improve urinary symptoms and prostate inflammation.

**Methods:**

One-hundred-twenty men with MetS waitlisted for BPH surgery were enrolled. They were categorized into *normal testosterone* (TT ≥ 12 nmol/L and cFT ≥ 225 pmol/L; *n* = 48) and *testosterone deficient* (TD) (TT < 12 nmol/L and/or cFT < 225 pmol/L; *n* = 72) then randomized to testosterone gel 2% (5 g/daily) or placebo for 24 weeks. At baseline and follow-up, questionnaires for urinary symptoms and trans-rectal ultrasound were performed. Prostate tissue was collected for molecular and histopathological analyses.

**Results:**

No differences in the improvement of urinary symptoms were found between TTh and placebo (OR [95% CI] 0.96 [0.39; 2.37]). In TD + TTh, increase in prostate but not adenoma volume was observed (2.64 mL [0.07; 5.20] and 1.82 mL [− 0.46; 0.41], respectively). Ultrasound markers of inflammation were improved. In a subset of 61 men, a hyper-expression of several pro-inflammatory genes was found in TD + placebo when compared with normal testosterone. TTh was able to counteract this effect. For 80 men, the inflammatory infiltrate was higher in TD + placebo than in normal testosterone (0.8 points [0.2; 1.4]) and TD + TTh men (0.9 points [0.2; 1.5]).

**Conclusions:**

Twenty-four weeks of TTh in TD men with BPH and MetS improves ultrasound, molecular and histological proxies of prostate inflammation. This does not result in symptom improvement.

**Supplementary Information:**

The online version contains supplementary material available at 10.1007/s40618-022-01776-9.

## Introduction

Benign prostatic hyperplasia (BPH)—also called benign prostate enlargement (BPE)—is impressively frequent in aging populations, with a 40–50% prevalence in men aged 50–60 years and up to 90% in men older than 80 years [1]. Despite its epidemiological relevance, the pathogenesis of BPH/BPE is still poorly understood. A new perspective suggested that metabolic derangement contributes, combined with aging, to BPH/BPE development and progression [1]. Recent studies indicate metabolic syndrome (MetS) as an early inducing and perpetuating factor for intraprostatic inflammatory process and overgrowth [2–8].

Immune tissue is well represented in the healthy prostate (prostate-associated lymphoid tissue; PALT) and it is crucial for the response to infectious pathogens coming from the urinary tract. After repeated stimulation, the activated PALT has the potential to set up a chronic, self-perpetuating inflammation with secretion of chemokines, cytokines and growth factors, eventually leading to stromal prostatic cell hyperplasia [9]. Besides being targets of bacterial or viral pathogens, stromal prostatic cells also behave as antigen‐presenting cells and activate antigen‐specific CD4^+^ T-cells, thus actively contributing to the organ‐specific inflammatory process [3, 4, 10, 11]. More importantly, human stromal cells from BPH tissue (hBPH) can respond not only to classical inflammatory stimuli, by expressing all toll-like receptors (TLRs), but also to several metabolic triggers (such as oxidized low-density lipoprotein; oxLDL, advanced glycated end-products; AGE, and insulin). This is supported also by an animal model of MetS induced by a high fat diet (HFD), in which the prostate develops BPH-like features [12]. As commonly observed in men, HFD animals develop hypogonadism and, interestingly, in vivo treatment with testosterone (T) prevents the occurrence of molecular and histological features resembling BPH [12]. Accordingly, in vitro pretreatment of hBPH with the selective androgen receptor (AR) superagonist dihydrotestosterone (DHT), blunted the inflammatory response induced by oxLDL [4]. In a multi-center retrospective study in BPH patients undergoing prostatectomy, we demonstrated that subjects with T deficiency (TD) have a higher intraprostatic inflammatory score than normal T men, with inflammatory scores progressively increasing as a function of lowering T levels [4].

It is conceivable that MetS triggers and maintains prostatic inflammation that could become chronic and result in prostatic hyperplasia (see [13] for review). Low T, which is a frequent finding in MetS, could exacerbate the inflammation, whereas testosterone therapy (TTh) may have beneficial effects (see [13] for review). Paradoxically, this working hypothesis substantially challenges the historical view of T as a foe for the prostate. In order to verify whether TTh in men with BPH, MetS and low T is able to improve lower urinary tract symptoms (LUTS) and intraprostatic inflammation, we designed a double blind, randomized clinical trial (RCT) providing TTh or placebo for 24 weeks to patients with the aforementioned characteristics on a waiting list for BPH surgery.

## Materials and methods

### Study participants

Adult men with BPH on the waiting list for surgery and that met the American Heart Association (AHA) and the National Heart, Lung, and Blood Institute (NHLBI) diagnostic criteria for MetS [14] were recruited. In order to study subjects with a significant inflammatory component of BPH, we included men with moderate prostatitis-like symptoms, as defined by an overall score on the National Institutes of Health Chronic Prostatitis Symptom Index (NIH-CPSI) > 15 [15]. Exclusion criteria were as follows: (i) Previous diagnosis, presence or suspected prostatic or breast cancer, (ii) prostate specific antigen (PSA) > 10 mg/mL, (iii) hematocrit > 52%, (iii) use of 5α-inhibitors in the previous three months, (iv) participation in another clinical trial, (v) presence of a serious organic or mental disease or other conditions that may affect the compliance to the study, and (vi) severe allergy or hypersensitivity to the study drug.

### Study design and protocol

The Florence PROTEST is a double-blind placebo-controlled RCT conducted at a single center at the Careggi Hospital—University of Florence. The study aimed to enroll 120 men. After signing the informed consent, all patients underwent the baseline visit procedures (V1). These included the NIH-CPSI [16] and the International Prostate Symptom Score (IPSS) [17] for the assessment of prostatitis-like symptoms and LUTS, respectively. For each patient, height, weight, waist circumference (WC) and blood pressure were measured; fasting blood samples were collected by 11 a.m. After having blood drawn, patients underwent a prostatic transrectal ultrasound (TRUS) with color Doppler method. Prostate was studied using the console MyLab Class C (Esaote SpA, Genova, Italy), scanning the organs at 5 mm intervals using a transrectal biplanar probe (linear transducer U533L 7.5 MHz; convex transducer U533C 6.5 MHz). To prevent bias, TRUS was performed intermittently by two experienced physicians (F.L. and S.C.) who were unaware of the clinical data and study arm. Prostate color-Doppler ultrasound features were defined as previously reported [18].

Total T, SHBG and PSA were measured (Modular E170 platform electrochemiluminescence immunoassays-Roche Diagnostics-Mannheim, Germany). The intra and inter-assay coefficients of variation for total T are 1.05% and 3.72% at 14.4 nmol/L. Based on total and free T [calculated (cFT) according to Vermeulen’s formula [19]], patients were classified into *normal T* (total T ≥ 12 nmol/L and cFT ≥ 225 pmol/L) or *testosterone deficient* (TD; total T < 12 nmol/L and/or cFT < 225 pmol/L). These thresholds are in accordance with the European Guidelines on functional hypogonadism [20, 21]. Since we were interested in the biological effect of T on prostate outcomes rather than the effect of TTh on TD symptoms, hypogonadism, defined by low T levels and consistent symptoms, was not an inclusion criterion. However, all but two men in the TD group were symptomatic based on the Aging Male Scale > 26 [22], International Index on Erectile Function < 22 [23] or presence of erectile dysfunction, impaired morning erections or low libido, as investigated by the ANDROTEST [24]. As soon as the hormone results became available (usually 2–3 days), TD men were randomized to receive T gel 2% (Tostrex, Kyowa Kirin S.r.l.) or placebo 5 g daily for 24 weeks. T or placebo gel was provided to patients together with accurate instructions on how to use it according to the Kiowa Kirin’s data-sheet. Afterwards, patients were seen after 24 weeks (V2). Over this period, patients received periodic telephone contacts to monitor medication compliance and possible adverse events. At V2, patients underwent the same procedures as at V1. After V2, patients were admitted to the Urology Unit within the following weeks to undergo planned surgery for BPH.

Transvesical prostatectomy was performed in 20 patients, whereas transurethral resection of the prostate (TURP) was performed in 60 patients. The quality of samples collected from TURP or transvesical prostatectomy is comparable [4]. Surgical specimens were obtained from at least three different sites of the adenomatous tissue.

As for routine procedure, most of the removed tissue was sent to the Pathological Anatomy Unit for histopathological assessment. Besides the routine evaluation, the inflammatory score was calculated [25], which scores the following characteristics of the infiltrate [4]: prevalent anatomical location (1 = stromal; 2 = periglandular; 3 = glandular), grade (1 = mild; 2 = moderate; 3 = severe) and extent (1 = focal; 2 = multifocal; 3 = diffuse).

Part of the harvested tissue was used for the assessment of inflammatory gene expression by quantitative RT‐PCR analysis using previously described methods [26]. Abbreviations for analyzed genes are reported in Table 1.

**Table 1:** Abbreviations for the genes analyzed by quantitative RT-PCR

Abbreviation	Extended gene name
Sex hormone receptors and androgen-dependent genes	
Androgen receptor	AR
Estrogen receptor α and β	ERα and ERβ
G protein-coupled estrogen receptor 1	GPER1
Progesterone receptor	PR
Prostate specific antigen	PSA
Acute phase response markers	
Monocyte chemoattractant protein-1	MCP-1
Cyclooxygenase-2	COX-2
Lactoferrin	LTF
Interleukin-1β, 6, 8	IL-1β, IL-6 and IL-8
T lymphocyte markers	
Toll-like receptor 2 and 4	TLR2 and TLR4
T-box transcription factor 21	TBX21
Retinoic acid receptor-related orphan receptor gamma (t)	RORγt
GATA binding protein 3	GATA3
Cluster differentiation 4 and 8	CD4 and CD8
Cytokines	
Interferon gamma-induced protein-10	IP-10
Interleukin-12A, 17, 4, 10	IL-12A, IL-17, IL-4 and IL-10
Metabolic receptors	
Oxidized low-density lipoprotein receptor-1	LOX-1
Receptor for advanced glycation endproducts	RAGE
Insulin receptor substrate 1	IRS1
Six-transmembrane protein of prostate 2	STAMP2
Fibroblastic differentiation	
Ras homolog family member A	RhoA
Rho-associated coiled-coil containing protein Kinase 1 and 2	ROCK1 and ROCK2

Further details on the protocol and methods are available as Supplementary materials.

Our hospital Independent Ethics Committee “Comitato Etico Area Vasta Centro—Azienda Ospedaliera Universitaria Careggi” approved this study on July 16th, 2012. All procedures were in accordance with the ethical standards of the institutional research committee and with the 1964 Helsinki Declaration and its later amendments. All participants provided written informed consent. All authors guarantee for the completeness and accuracy of the data and analyses. All authors reviewed the manuscript and approved it for contents, accuracy and consistency with the study protocol. Kiowa Kirin provided Tostrex and placebo without any cost but had no part in the study design, data analysis and interpretation or manuscript draft. The study was registered at the clinicaltrials.gov website (NCT02366975).

### Randomization procedure and blinding

Patients were centrally randomized 1:1 using an interactive web-response system and the minimization algorithm. Randomization was stratified by age < / ≥ 45 years. Once classified as TD, a randomization number was assigned to patients, which corresponded to a set of gel packages covering the entire study period. T gel and placebo presented as indistinguishable packages identified only by a number corresponding to the random number assigned to each patient. Patients, study personnel performing visit procedures, and personnel involved in laboratory procedures or data analysis were all blinded to the treatment arm. In addition, personnel performing laboratory analysis on prostate tissue was blinded to clinical data.

### Outcome measures

**Fig. 1:**
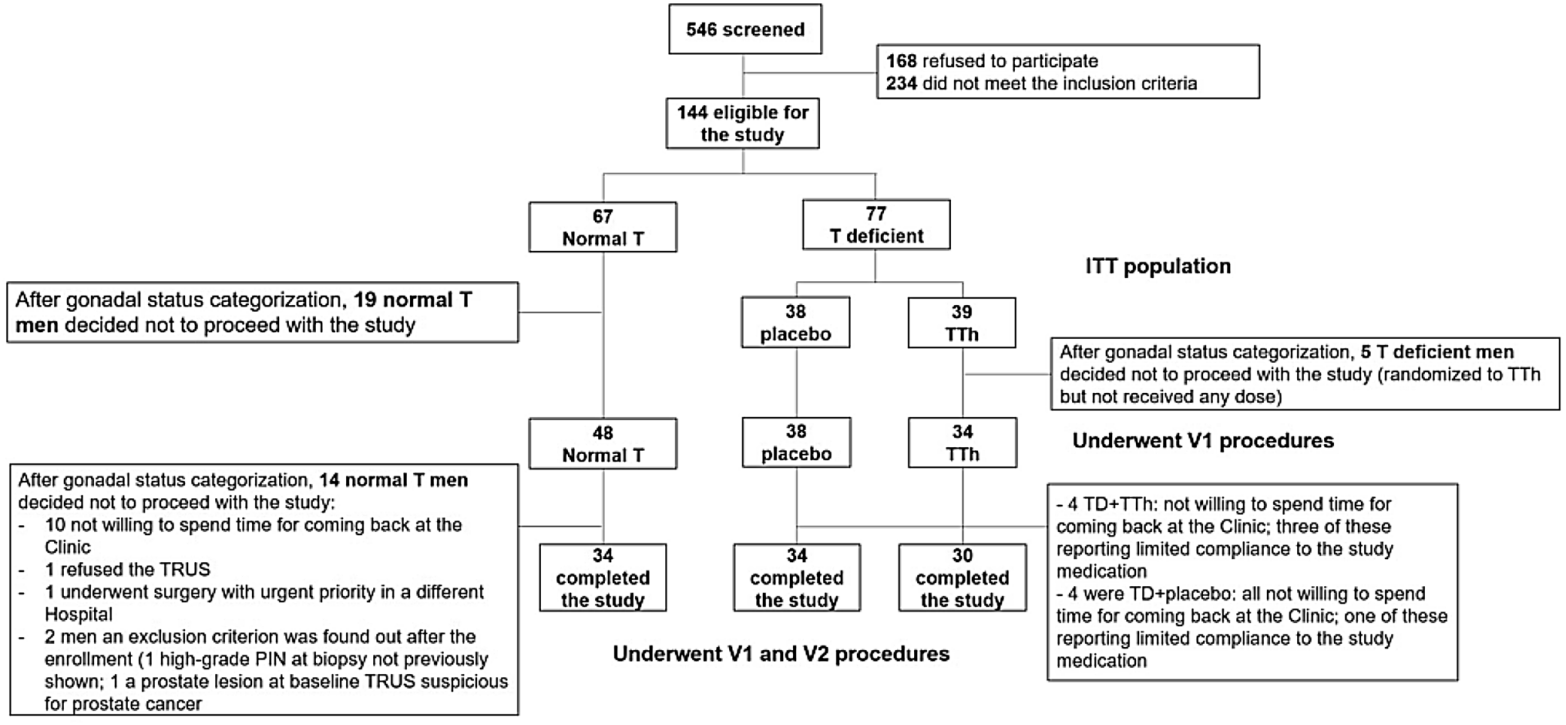
Study flow-chart. *TTh* testosterone therapy; *ITT* intention-to-treat

The primary outcome was the improvement over 24 weeks of prostatitis symptoms and LUTS in TD men with MetS and BPH treated with TTh. Improvement was defined by at least two of the following: (i) reduction by at least 2 points of the NIH-CPSI score, (ii) reduction by at least 3 points of *total IPSS* score, and (iii) reduction by at least 1 point in the *IPSS bother* score. The primary analysis was based on the intention to treat (ITT) population. Patients that were randomized but did not have either V1 or V2 evaluation were set as “not improved”. Sensitivity analyses were performed restricting the analysis to men having only V1 questionnaires or both V1 and V2 questionnaires (see Fig. 1).

Secondary outcomes were as follows: (1) questionnaire scores (IPSS total score, IPSS bother score, NIH-CPSI total score), (2) blood parameters (PSA, hemoglobin, and hematocrit), (3) TRUS parameters (prostate diameters and volume, prostate adenoma diameters and volume, prostate arterial peak systolic velocity and acceleration), (4) inflammatory gene expression in prostatic tissue collected at surgery (see Table 1 for genes evaluated), and (5) histopathological inflammatory score in prostatic tissue collected at surgery. Secondary outcomes were evaluated as continuous variables. Those measured at V1 and V2 (points 1–3 of the list above) were assessed as change between and within groups over time. For parameters measured only at follow-up (points 4 and 5 of the list above), the difference between groups was evaluated. Only men who eventually underwent surgery at our Hospital (*n* = 80) entered the histology analysis and those for which an extra prostate sample was available (*n* = 61) were included in the molecular analysis.

### Statistical analysis

The study was designed to have an 80% power at an alpha level of 0.05, with the use of two-tailed chi-square statistical test to detect an absolute difference in the rate of improvement between the TD groups of 25%. This required 38 participants per TD arm. Since we were interested in recruiting also men with normal T as a comparison group for the analyses on the prostate samples, we planned to recruit overall 120 men with MetS and BPH. At 120 patients enrolled, 63 were TD and we proceeded with the recruitment until the number of TD patients was achieved.

The dependence upon TTh of the improvement in urinary symptoms in TD subjects was assessed by the odds ratio (OR), and the significance of the difference in the proportion of improvement in the two treatment groups was evaluated with the Chi-square test, using the TD + placebo arm as the comparator. For the analyses on secondary outcomes, the change over time in the two treatment groups was assessed by the multilevel mixed-effects linear regression using the treatment arm, the time-points and their interaction as independent variables, the measure of the variable at V2 as the dependent variable and the patients’ ID as random effect. The analyses were adjusted for the V1 value of the dependent variable along with age, WC, and total T at V1. For each outcome, the contrast between the TD groups at V2 [estimated marginal means (EMM) and 95% confidence interval (CI) with the p-value between groups (p_between_)] were reported. In addition, the EMM and 95% CI for the change over time within each TD arm were reported with the p-value within each group (p_within_). For measures on prostate samples, for which V1 value was not available, the normal T group was used as the control for the TD groups. The differences among the three groups were assessed by unpaired two-tailed Kruskal–Wallis test, followed by Mann–Whitney two sample statistic, for non-normally distributed parameters (gene expression) or one-way ANOVA test with post-hoc analysis (Bonferroni) for normally distributed variables (i.e. inflammatory score). All reported *p* values are two-sided and values < 0.05 were considered statistically significant. All statistical analyses were conducted using Stata MP 13.1 for Windows (StataCorp, College Station, TX, USA).

## Results

### Recruitment, participants and retention

The recruitment started on November 1st 2012 and closed on June 23rd 2016. Figure 1 reports the study flow-chart. After the randomization, 120 men continued with the study procedures: 48 had normal T and 72 were TD. Of the latter, 34 were assigned to TTh, whereas 38 were assigned to placebo. Twenty-two men did not complete the V2 procedures but maintained their consent to include their data and collect prostate tissue at surgery. Of these, 14 men had normal T levels (10 were not willing to spend the time to come back to the Clinic, one refused the TRUS, one underwent surgery with urgent priority in a different Hospital, for two men an exclusion criterion was found out after the enrollment because one showed a previous biopsy reporting a diagnosis of high-grade prostatic intraepithelial neoplasia (high-grade PIN) and one had a prostate lesion at V1 TRUS suspicious for prostate cancer), four were TD + TTh (all not willing to spend the time to come back to the Clinic and three of these reported limited compliance to the study medication) and four were TD + placebo (all not willing to spend the time to come back to the Clinic and one reported limited compliance to the study medication).

The characteristics of the analytical sample at V1 are reported in Table 2. No modifications in medications occurred over the trial period.

**Table 2:** Baseline characteristics of the sample

	Normal T (1)	T-deficient + Placebo (2)	T-deficient + TTh (3)	*p* 1 vs. 2	*p* 1 vs. 3	*p* 2 vs.3
*N*	48	38	34	–	–	–
Age (years)	67.8 ± 7.6	69.6 ± 6.2	67.8 ± 7.1	0.243	0.999	0.283
Total T (nmol/L)	17.3 ± 4.1	10.6 ± 4.1	10.1 ± 4.3	** < 0.0001**	** < 0.0001**	0.607
SHBG (nmol/L)	48.9 ± 14.7	45.7 ± 20.4	46.9 ± 18.7	0.425	0.646	0.778
cFT (pmol/L)	281.4 ± 47.4	167.7 ± 44.1	164.3 ± 31.2	** < 0.0001**	** < 0.0001**	0.752
Hct (%)	44.8 ± 2.2	43.4 ± 3.2	43.1 ± 3.9	0.085	**0.050**	0.743
Hb (g/dL)	15.2 ± 0.9	14.7 ± 1.3	14.4 ± 1.2	0.096	**0.020**	0.462
PSA (ng/mL)	1.9 [1.2–3.3]	2.4 [1.3–4.4]	1.6 [0.9–3.0]	0.407	0.730	0.260
Total cholesterol (mg/dL)	198.1 ± 41.1	194.1 ± 35.6	188.5 ± 194.1	0.693	0.369	0.609
HDL-cholesterol (mg/dL)	45.8 ± 10.3	51.3 ± 16.8	49.3 ± 48.6	0.091	0.301	0.574
Triglycerides (mg/dL)	113.0 [97.0–152.0]	107.0 [81.0–155.0]	108.0 [74.0–155.0]	0.395	0.265	0.790
Waist circumference (cm)	105.9 ± 10.5	105.7 ± 10.8	108.3 ± 11.9	0.946	0.358	0.343
Systolic blood pressure (mmHg)	130.0 [122.5–140.0]	130.0 [130.0–140.0]	135.0 [130.0–140.0]	0.480	0.819	0.664
Diastolic blood pressure (mmHg)	80.0 [80.0–85.0]	80.0 [76.3–85.0]	80.0 [80.0–87.5]	0.406	0.546	0.856
Antihypertensive drugs (%)	64.4	66.7	56.3	0.834	0.467	0.378
Hypolipidemic drugs (%)	46.7	44.4	43.8	0.842	0.800	0.954
Antidiabetic drugs (%)	13.3	27.8	25.0	0.105	0.191	0.796
Alpha-blockers (%)	75.0	70.0	82.4	0.169	0.247	0.260
NIH-CPSI	19.3 ± 4.3	19.1 ± 4.9	19.5 ± 5.9	0.807	0.884	0.716
IPSS total score	21.2 ± 7.5	17.6 ± 8.4	17.1 ± 9.8	0.061	**0.036**	0.787
IPSS bother	2.6 ± 1.6	2.6 ± 1.6	2.8 ± 1.6	0.942	0.560	0.625
Prostate transverse diameter (mm)	108.2 ± 15.3	104.7 ± 15.4	93.2 ± 14.7	0.920	0.673	0.754
Prostate longitudinal diameter (mm)	56.9 ± 7.4	56.1 ± 10.2	55.3 ± 7.1	0.686	0.413	0.684
Prostate anteroposterior diameter (mm)	39.2 ± 9.5	41.2 ± 8.7	38.4 ± 9.5	0.381	0.713	0.237
Prostate adenoma transverse diameter (mm)	45.8 ± 12.7	46.8 ± 9.3	45.9 ± 13.2	0.775	0.982	0.795
Prostate adenoma longitudinal diameter (mm)	49.13 ± 10.1	48.7 ± 7.7	47.8 ± 10.5	0.848	0.557	0.699
Prostate adenoma anteroposterior diameter (mm)	35.5 ± 10.7	37.6 ± 8.8	35.6 ± 9.5	0.352	0.959	0.400
Prostate volume (mL)	71.3 ± 32.3	75.0 ± 27.7	68.7 ± 35.8	0.622	0.725	0.417
Prostate adenoma volume (mL)	50.6 ± 36.1	49.5 ± 24.2	51.2 ± 34.6	0.906	0.951	0.857
Prostate arterial peak systolic velocity (cm/s)	17.2 ± 6.4	17.2 ± 4.4	17.3 ± 5.6	0.994	0.951	0.946
Prostate arterial acceleration (m/s^2^)	119.1 ± 66.1	108.4 ± 35.2	104.8 ± 34.0	0.356	0.235	0.774

Data are reported as mean ± standard deviation, median [interquartile range] or percentage for normally distributed, non-normally distributed or categorical variables, respectively

*p* values denote the significance level for the comparison between groups as derived from one-way ANOVA test with post-hoc analysis

*TTh* testosterone therapy; *T* testosterone; *SHBG* sex hormone binding globulin; *cFT* calculated free testosterone; *Hct* hematocrit; *Hb* hemoglobin; *PSA* prostate specific antigen; *HDL* high-density lipoprotein; *NIH*-*CPSI* National Institutes of Health Chronic Prostatitis Symptom Index; *IPSS* International Prostate Symptom Score

### Change in testosterone and SHBG levels

As expected, at V2, total and free T levels were significantly increased in the TD + TTh arm compared to the TD + placebo men (Table 3).

**Table 3:** Change over time in secondary outcomes and safety parameters in the TD + TTh and TD + placebo study arms

	TD + placebo			TD + TTh			TD + TTh vs. TD + placebo
	V1	V2	Contrast within group	V1	V2	Contrast within group	Contrast between groups at V2
Symptom score							
IPSS total score	15.49 [13.74;17.25]	14.78 [12.96;16.59]	− 0.72 [− 2.88;1.44] *p* = 0.514	15.40 [13.43;17.37]	15.69 [13.72;17.66]	0.29 [− 2.07;2.65] *p* = 0.809	0.92 [− 1.78;3.61] *p* = 0.506
IPSS bothering score	2.88 [2.48;3.29]	2.75 [2.32;3.18]	− 0.13 [− 0.72;0.45] *p* = 0.659	2.94 [2.47;3.41]	2.25 [1.78;2.73]	**− 0.69** **[− 1.35;− 0.03]** ***p* = 0.042**	− 0.50 [− 1.14;0.15] *p* = 0.131
NIH-CPSI	18.63 [16.92;20.33]	14.48 [12.65;16.32]	**− 4.14** **[− 6.63;− 1.65] *p* = 0.001**	18.88 [16.97;20.79]	14.15 [12.17;16.14]	**− 4.73** **[− 7.47;− 1.99]** ***p* = 0.001**	− 0.33 [− 3.05; 2.38] *p* = 0.810
Prostate diameters (mm)							
Transverse	88.77 [66.63–110.90]	61.89 [39.05–84.74]	− 26.88 [− 58.45;4.69] *p* = 0.095	83.25 [59.53–106.96]	59.14 [33.61–84.67]	− 24.11 [− 58.64;10.43] *p* = 0.171	− 2.75 [− 37.32;31.83] *p* = 0.876
Longitudinal	55.37 [54.12–56.62]	56.42 [55.13–57.71]	1.05 [− .73;2.83] *p* = 0.249	55.55 [54.21–56.90]	56.12 [54.68–57.57]	0.57 [− 1.38;2.52] *p* = 0.568	− 0.29 [− 2.25;1.66] *p* = 0.769
Anteroposterior	39.75 [39.10–40.40]	40.00 [39.33–40.67]	0.25 [− 0.68;1.17] *p* = 0.600	39.87 [39.18–40.57]	41.15 [40.40–41.90]	**1.28** **[0.27;2.29]** ***p* = 0.013**	**1.15** **[0.13;2.17]** ***p* = 0.027**
Prostate adenoma (mm)							
Transverse	46.58 [45.79–47.37]	46.23 [45.42–47.04]	− 0.35 [− 1.47;0.78] *p* = 0.548	46.53 [45.65–47.42]	47.21 [46.18–48.24]	0.67 [− 0.68;2.03] *p* = 0.329	0.97 [− 0.35;2.30] *p* = 0.148
Longitudinal	47.44 [46.56–48.32]	48.04 [47.14–48.95]	0.60 [− 0.65;1.86] *p* = 0.344	47.39 [46.45–48.33]	47.52 [46.51–48.53]	0.13 [− 1.24;1.50] *p* = 0.853	− 0.52 [− 1.89;0.85] *p* = 0.454
Anteroposterior	36.28 [35.73–36.84]	36.21 [35.64–36.77]	− 0.08 [− 0.86;0.70] *p* = 0.845	36.35 [35.76–36.95]	37.26 [36.59–37.92]	**0.90** **[0.02;1.79]** ***p* = 0.045**	**1.05** **[0.16;1.93]** ***p* = 0.020**
Prostatic arterial blood flows							
Arterial peak systolic velocity (cm/s)	16.74 [15.83–17.66]	16.33 [15.37–17.29]	− 0.41 [− 1.73;0.91] *p* = 0.540	16.98 [16.01–17.96]	14.67 [13.62–15.75]	**− 2.32** **[− 3.74;− 0.89]** ***p* = 0.001**	**− 1.67** **[− 3.10;− 0.23]** ***p* = 0.023**
Acceleration (m/s^2^)	103.80 [96.49–111.15]	102.43 [94.79–110.08]	− 1.37 [− 11.88;9.15] *p* = 0.799	106.22 [98.39–114.05]	89.26 [80.38–98.14]	**− 16.96** **[− 28.70;− 5.22]** ***p* = 0.005**	**− 13.18** **[− 24.96;− 1.39]** ***p* = 0.028**
Hormone and binding hormone parameters							
Total Testosterone (nmol/L)	10.84 [9.13;12.56]	12.81 [10.98;14.64]	1.97 [− 0.53;4.46] *p* = 0.123	10.57 [8.66;12.48]	16.60 [14.66;18.55]	**6.03** **[3.32;8.74]** ***p ***<** 0.0001**	**3.79** **[1.11;6.47]** ***p* = 0.006**
Calculated free testosterone (pmol/L)	169.73 [128.80;210.66]	196.74 [149.88;243.60]	27.00 [− 34.62;88.63] *p* = 0.390	158.03 [111.89;204.175]	308.12 [258.06;358.18]	**150.09** **[82.72;217.45]** ***p ***<** 0.0001**	**111.38** **[42.17;180.59]** ***p* = 0.002**
SHBG (nmol/L)	48.60 [44.86;52.35]	52.14 [47.84;56.43]	3.53 [− 2.13;9.20] *p* = 0.222	48.57 [44.35;52.78]	46.39 [41.93;50.85]	− 2.18 [− 8.26;3.90] *p* = 0.483	− 5.74 [− 11.99;0.50] *p* = 0.072
Safety parameters							
Hematocrit (%)	43.26 [42.48;44.04]	41.09 [40.19;41.99]	**− 2.17** **[− 3.36;− 0.99]** *p *< **0.0001**	43.25 [42.33;44.16]	43.92 [42.58;45.26]	0.67 [− 0.93;2.27] *p* = 0.411	**2.83** **[1.20;4.46]** ***p* = 0.001**
PSA (ng/mL)	1.99 [1.62;2.46]	1.81 [1.46;2.25]	0.91 [0.71;1.16] *p* = 0.438	1.94 [1.57;2.40]	2.25 [1.82;2.78]	1.16 [0.91;1.47] *p* = 0.228	1.24 [0.92;1.69] *p* = 0.164

Data are derived from multilevel mixed-effects linear regression. Results are reported as estimated marginal means (EMM) and 95% confidence interval at V1 (baseline) and V2 (after 24 weeks) in the TD study arms. Contrast in EMM and 95% confidence interval within the same group and between groups at V2 are also reported. Bold data are intended to highlight statistical significant changes

*TD* testosterone-deficient; *TTh* testosterone therapy

Efficacy: clinical and instrumental parameters

#### LUTS and prostatitis-like symptoms

In both the TD groups, 16 men reported an improvement. When considering the ITT population, the success rate was 41.03% (16 out of 39 TD + TTh men) vs. 42.11% (16 out of 38 TD + placebo men). The OR for a symptomatic improvement in TD + TTh when compared to TD + placebo was 0.96 [0.39–2.37] (*p* = 0.923). When excluding 5 men randomized to TTh but never treated with the study medication (decided not to proceed with the study), the results obtained did not change being the success rate among the TD + TTh and TD + placebo men 47.06% and 42.11%, respectively (OR 1.22 [0.48–3.10], *p* = 0.673). When considering only men who underwent the V2, similar results were obtained (OR 1.13 [0.42–3.01], *p* = 0.814), with 15 successes out of 30 TD + TTh men (50.00%) and 16 out of 34 TD + placebo men (47.06%).

Table 3 shows the change of the questionnaire scoring in the treatment groups over time. At V2, the IPSS total score was not different between the TD groups. The IPSS bothering score significantly decreased over time in the TD + TTh arm but not in the TD + placebo one, although the difference at V2 between the TD groups did not achieve statistical significance. The NIH-CPSI score significantly declined in both groups with a similar trend of not being different at V2 in the TD groups.

#### TRUS

The change in prostatic diameters over time in the TD arms is shown in Table 3. At V2, TD + TTh and TD + placebo did not differ in transverse and longitudinal diameters, whereas the anteroposterior diameter was slightly longer in the TD + TTh arm than in the TD + placebo one. Similar results were observed for the prostate adenoma diameters. When the ellipsoid volume formula was applied, the difference in EMM at V2 between the two study arms was 2.64 mL [0.07; 5.20], *p* = 0.044 for the prostate volume and 1.82 mL [− 0.46; 0.41], *p* = 0.119] for the adenoma volume.

The assessment of the change in the prostatic artery blood flows (Table 3) showed at V2 a significantly decreased peak systolic velocity (PSV) and acceleration in the TD + TTh arm compared to the TD + placebo group.

### Efficacy: molecular and histological parameters

#### mRNA expression of the hormone receptor, inflammatory- and insulin sensitivity-related genes in the prostate

**Fig. 2:**
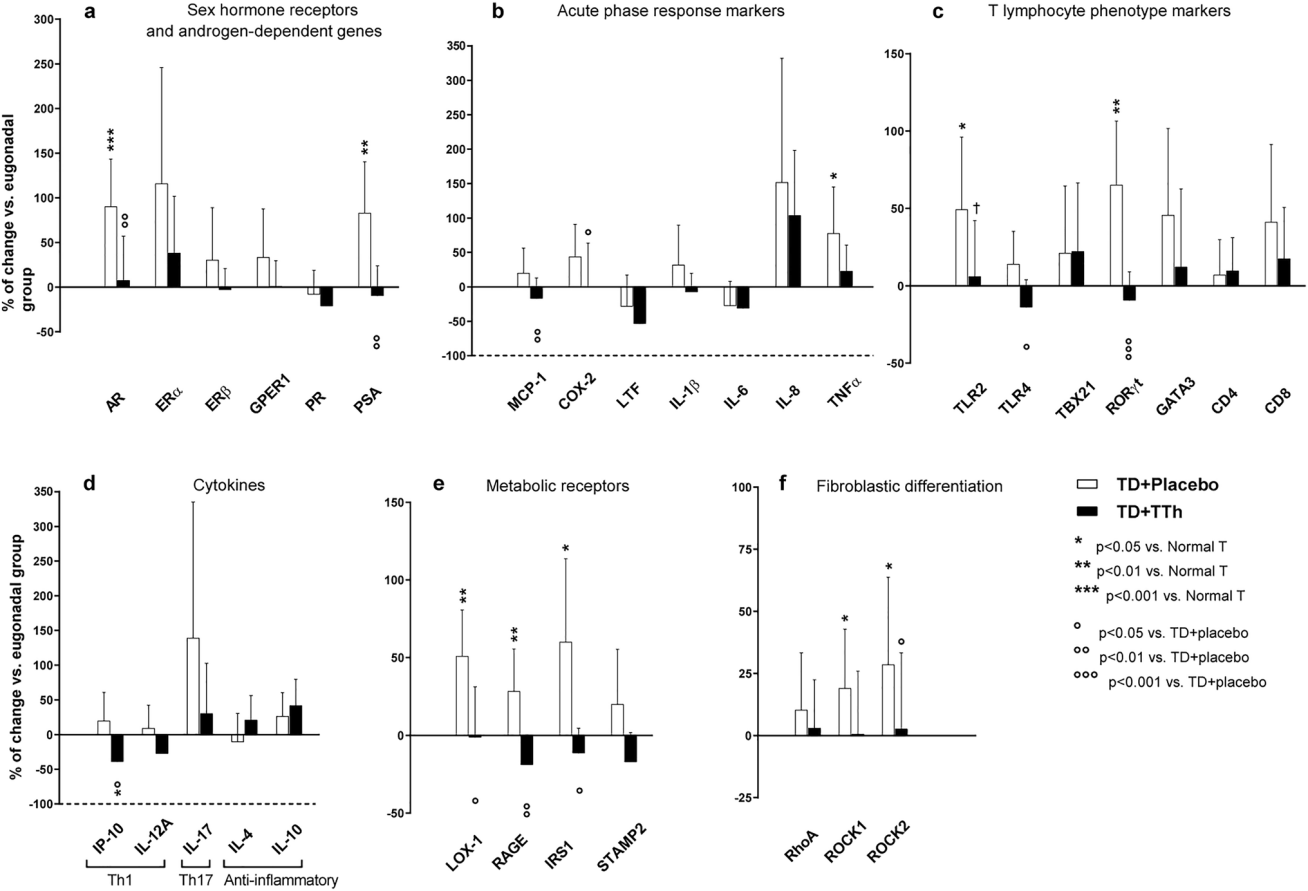
Panels **A**–**F**: Expression in prostatic tissue from TD + placebo and TD + TTh men (being normal T the referent) of genes involved in different phases of inflammatory and immune response, genes encoding for metabolic receptors and genes involved in fibroblastic trans-differentiation. Data were calculated according to comparative *C*_t_ method by rRNA subunit 18S as the reference gene for normalization. Results derived from unpaired two-tailed Kruskal–Wallis test, followed by Mann–Whitney two-sample statistic and are expressed in percentage over control and are reported as means and 95% confidence interval. Meaning of symbols is reported in figure as inset. Abbreviations for analyzed genes are reported in Table 1

For 25 normal T, 19 TD + placebo and 17 TD + TTh men, the prostate tissue could be harvested at surgery for molecular analyses. When compared to the normal T arm, TD + placebo had an increased expression of several hormone receptors and PSA (Fig. 2, Panel A) as well as of several genes involved in different steps of the inflammatory and immune process (Fig. 2, Panels B-D). Among cytokines, besides an increase in pro-inflammatory ones, a decrease in the anti-inflammatory cytokine IL-4 was found.

The TD + placebo arm was characterized by a marked increase in metabolic receptors, including LOX-1, RAGE, IRS1 and STAMP2 (Fig. 2, Panel E) and by an increased expression of RhoA, ROCK1 and ROCK2 that marks a fibroblastic trans-differentiation (Fig. 2, Panel F). Despite not all the aforementioned differences between TD + placebo and normal T men achieving statistical significance, a clear trend was well recognizable for most of these (Fig. 2).

TTh counteracted most of the upregulations observed in the TD + placebo group. In particular, the expression of AR, PSA, TLR4, RORγt, LOX-1, RAGE, IRS1 and ROCK2 was significantly decreased compared to the TD + placebo arm (Fig. 2). Further inflammation-related genes were significantly less expressed in TTh than in placebo-treated men, including MCP-1, COX-2, and IP-10. In the TD + TTh group, the expression of IP-10 was significantly reduced below that of men with normal T and a trend towards significance was found for MCP-1 (Fig. 2, Panels B and D). It should be also underlined that, even when the difference was not statistically significant, in the TD + TTh group an opposite trend than in the TD + placebo group was observed for several key genes, suggesting a reduced expression upon TTh of pro-inflammatory genes (i.e. IL-1β, IL-8, TNFα, LTF, GATA3, CD8, IL-17), and an upregulation of anti-inflammatory ones (IL-4 and IL-10). Similarly, in the TD + TTh arm, the expression of ER and PR tended to be lower than in the TD + placebo arm, as well as the expression of STAMP2, RhoA, ROCK1 and ROCK2.

The exclusion from the analysis of men with incidental high-grade PIN or adenocarcinoma did not substantially change the results (not shown).

#### Histopathological inflammatory score

**Fig. 3:**
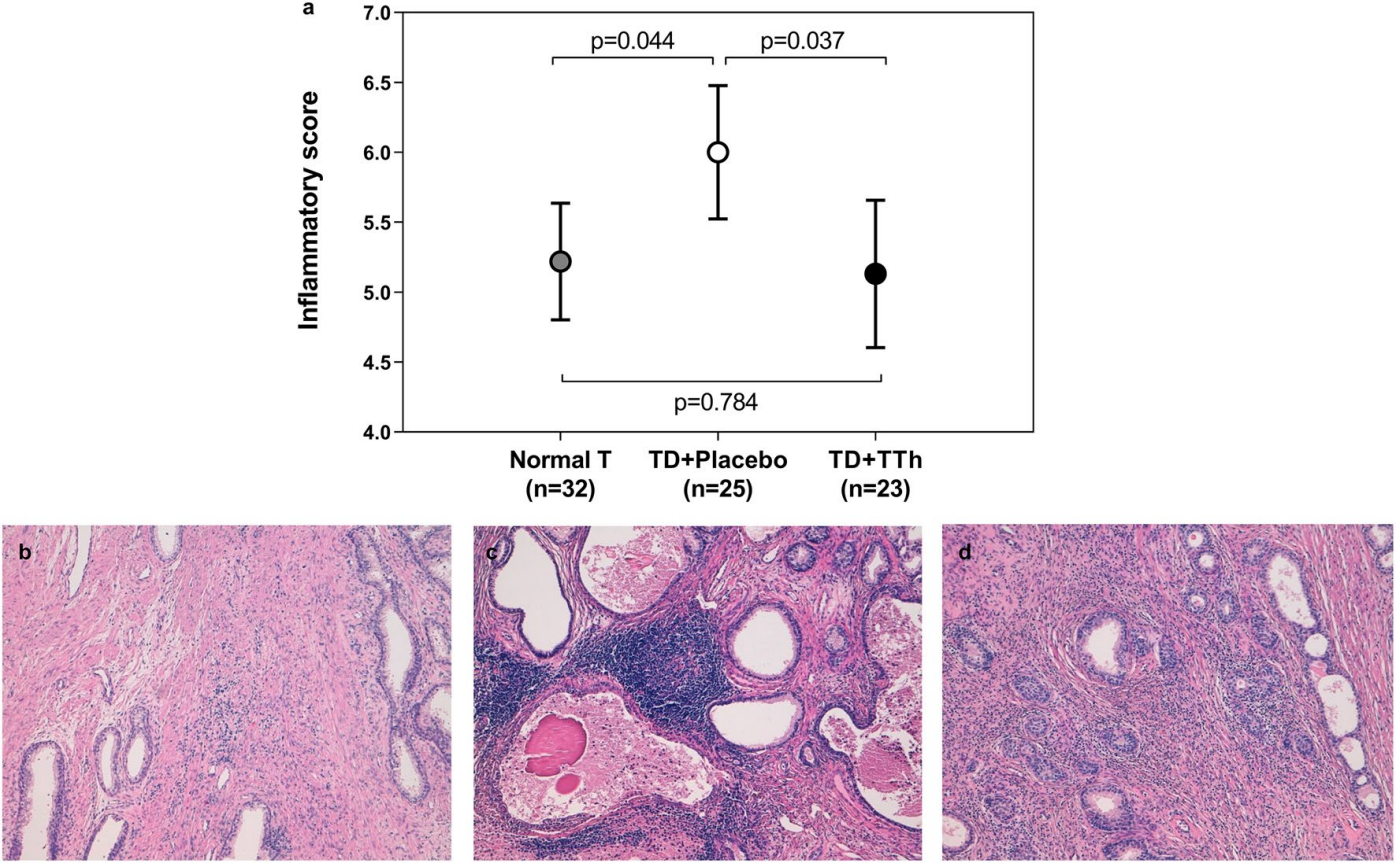
Panel A: Inflammatory score evaluated in prostatic samples from normal T, TD + placebo and TD + TTh men. Results derive from one-way ANOVA test with post-hoc analysis and are reported as means and 95% confidence interval. Panel B-D: Histopathological features of prostatic inflammation; Panel B (normal T patient): Scant inflammatory infiltrate within prostatic BPH stroma; Panel C (TD + placebo patient): Confluent sheets of inflammatory cells with follicle formation centered on glands and involving glandular epithelium and lumen. Panel D (TD + TTh patient): Mild stromal inflammation sparing hyperplastic prostatic glands. *TD* Testosterone deficiency, *TTh*  testosterone therapy, *BPH* benign prostatic hyperplasia

In 80 men (32 normal T, 25 TD + placebo and 23 TD + TTh) who underwent surgery at our research clinic, the inflammatory score was calculated. As shown in Fig. 3 Panel A, TD + placebo men had significantly higher inflammatory scores than those with normal T. TTh significantly reduced the inflammatory score to values comparable with normal T men. Figure 3 Panels B–D report three examples of prostatic tissue from each study group. When metabolic parameters or medications were considered, none of them but hypoglycemic medications showed a significant effect as modulating factors on the observed change of IS (Supplementary Table 1). Other sub-analyses focused on specific categories of medications were not possible due to the limited number of patients in each subgroup.

The exclusion of men with incidental high-grade PIN or adenocarcinoma did not affect substantially the results (not shown).

### Safety parameters

At V2, hematocrit was significantly higher in TD + TTh than TD + placebo group (Table 3). Only one subject had hematocrit above 50% (i.e. 50.7%) at V2 and he was in the TD + TTh group.

Circulating PSA was not different between the groups (Table 3). PSA levels above 4.0 ng/mL were found in 7 out of 31 and 8 out of 30 subjects (22.58 vs. 26.67%, *p* = 0.294) in the TD + placebo and TD + TTh arm, respectively.

Overall, 9 incidental prostatic neoplasia (one high-grade PIN and 8 adenocarcinomas) were found at the histology inspection. The adenocarcinomas were equally distributed in the study groups, with 4 in the normal T (12.50%), 2 in the TD + placebo (8.00%) and 2 in the TD + TTh group (8.70%) (*X*^2^ = 2.86 *p* = 0.581). The high-grade PIN was found in a man from the TD + TTh group.

## Discussion

This is the first double-blind, placebo-controlled RCT providing data on the effects of TTh on prostate health in TD men with BPH and MetS, taking into account clinical, instrumental, histological and molecular parameters. By using this multiparametric approach, we demonstrate that TTh—administered for 24 weeks in men with low T and MetS and a candidate for prostate surgery for BPH—is a safe option and it could even be beneficial. In fact, this study is the first to substantiate within the human prostate an anti-inflammatory role of TTh administered in vivo. This is demonstrated by the reduction of color-Doppler ultrasound, histological and molecular markers of inflammation in the prostate collected from the group of TD men treated for 24 weeks with TTh. Moreover, despite a slight increase in total prostate volume, we did not observe any significant increase of adenoma volume or worsening in LUTS.

The aforementioned findings are pursuant to previous results obtained by our group in a rabbit model of MetS induced by a HFD. In this animal model, besides metabolic derangements and hypogonadotropic hypogonadism, several prostatic histological alterations resembling BPH developed i.e. tissue remodelling with hypoxia, fibrosis and a chronic and self-perpetuating (Th)1/Th17 inflammatory process [12]. In HFD rabbits, the in vivo administration of T counteracted all the aforementioned intraprostatic alterations including the inflammatory process [12]. Similarly, in prostate tissue from TD men with MetS, the expression of several inflammatory factors was higher than in normal T men. In particular, in the TD + placebo arm, typical markers of the innate immune response (TNFα, MCP-1, IL-1β, IL-6, IL-8 and COX-2), of the Th1- (TBX21, IP-10, IL-12A) and Th17-response (RORγt and IL-17) were more expressed than in the normal T group. The induction of a Th1/Th17 phenotype represents the tipping point from an acute inflammatory response towards a chronic autoimmune inflammation, which is a prominent pathogenic process in BPH development and progression. Accordingly, an increased mRNA expression of either TLR2 or TLR4 (cell surface markers of either immune or non-professional antigen presenting cells, such as stromal prostatic cells [9])—and lymphocyte surface markers (CD4 and CD8) were observed. In contrast, the associated increase of Th2-response (marked by GATA3), might be explained as a counter-regulatory attempt against the self-perpetuating Th1/Th17-driven inflammation [1, 9, 27, 28]. The prostate from TD patients also showed an increased expression of several metabolic receptors [4, 11], such as LOX-1, RAGE, IRS1 and STAMP2, and fibroblast trans-differentiation genes (RhoA/ROCK pathway), all of which exert important biological functions within metabolic, inflammatory and mitogenic/growth promoting pathways [29–34].

Collectively, these data suggest that, in the prostate of men with low T, severe inflammatory and tissue remodelling pathways are operating. Noteworthy, hereby we demonstrate that almost all the modifications observed in gene expression from the prostate of TD men treated with placebo are improved by TTh. A resolution of inflammation is also demonstrated by the significant increase in two of the most well-known anti-inflammatory cytokines, IL-10 and IL-4, besides the aforementioned inflammatory and tissue-remodeling genes [35, 36]. Hence, our results show that TTh in the human prostate has an inhibitory effect on several steps of the pathway that leads to the development of a chronic, self-perpetuating inflammatory condition.

A point that deserves further discussion is the down-regulation of genes related to meta-inflammatory receptors [4, 11], including LOX-1, RAGE, IRS1 and STAMP2, in the prostate from TTh-treated patients compared to placebo-treated TD men, reaching levels that were even statistically lower that those detected in normal T ones. Pre-treating BPH stromal cells with DHT induced the anti-inflammatory cytokine IL-10 and suppressed LOX-1 expression [4], thus blunting the ability of metabolic factors to trigger the secretion of proinflammatory cytokines (i.e. IL-8 and IL-6), as hereby observed in the prostate from TTh subjects.

The modulation of the expression of PSA within the prostate tissue is interesting and unexpected. PSA production is notoriously androgen-dependent [37]; however, PSA is also regulated by inflammatory factors [38] thus justifying the elevated PSA levels detected in men with prostatitis [39, 40]. In this view, the increased PSA expression in the prostate of TD + placebo men, decreased by TTh, could be considered further evidence for the role of T in counteracting the intraprostatic inflammatory process. Noteworthy, the molecular findings are confirmed by the increased histological inflammatory features within the prostate of TD men with MetS that are restored by TTh to levels observed in men from the normal T group.

The Doppler TRUS assessment further corroborated this evidence. The enhanced vascularization and arterial PSV are indeed proven markers of intraprostatic inflammation [18].

The improvement in prostatic inflammation contrasts with the slight increase of prostate volume observed in the TD + TTh arm. However, this inconsistency is only apparent because prostate growth and prostate inflammation are two distinct events, not necessarily related. The androgen dependence of the prostate growth is expected, considering the role of androgens in prostate development and growth during fetal and peripubertal life [13]. Accordingly, 5α-reductase inhibitors are effectively used in BPH to decrease prostate volume. However, the Prostate Cancer Prevention Trial showed that men treated for 7 years with finasteride, although having significantly smaller prostate volume, had a higher prevalence and extent of intraprostatic inflammation compared to placebo [41, 42]. Our results are consistent with these data, showing a TTh-dependent increase in the prostate volume and a concomitant decrease in inflammation.

The objective improvement in prostate inflammation is not accompanied by a subjective improvement in LUTS. Despite this finding failing our initial hypothesis upon which the present RCT was designed, this data reassures us about the safety of 24 weeks of TTh even in BPH men who are candidates for surgery, if low serum T is documented. A possible explanation for the lack of improvement in LUTS is that 24 weeks of treatment are not sufficient for detecting a clinically relevant change. Indeed, similar to our study, no change in LUTS upon TTh was found by previous placebo-controlled RCTs [43], none of these with follow-ups longer than 1 year. A discrepancy in the period needed for achieving an improvement in subjective or objective measures has been previously documented for medications used in BPH. In the Medical Therapy of Prostatic Symptoms trial, after just 1 year of treatment, finasteride produced a significant amelioration in urinary flow rate and prostatic volume in comparison with placebo, but the symptomatic relief was observed only after 4 years [44]. Thus, it could also be hypothesized that TTh requires a longer time for improvement in symptoms. It could be argued that longer-term treatments could even unveil a worsening in LUTS but the slight improvement in bothering for LUTS detected in the TTh arm may suggest an initial trend towards a subjective relief in LUTS.

Besides reassurance on the safety of TTh on LUTS in TD men with MetS, candidates for surgery for BPH, this study also provides comforting evidence on other sensitive safety issues. In fact, this RCT, which excluded men with suspicious or actual prostate cancer, demonstrated that there is no difference in rate of incidental prostate cancer in men undergoing or not TTh. Only one moderate increase in hematocrit was detected and, as expected, it was found in a TTh treated patient.

Strengths are represented by the double-blind placebo-controlled study design. In addition, the collection of prostate tissue at surgery allowed for the performing of the first RCT evaluating the effects of TTh on the inflammatory features of BPH. In fact, although a RCT evaluating the effect of TTh or placebo on molecular and histological parameters derived from prostatic tissue in TD men has been previously published [45], this was mainly focused on prostate cancer risk, rather than on inflammation. A further strength is represented by the multiparametric assessment of prostatic inflammation, which produced consistent results and corroborated the reliability of our conclusions.

Limitations are represented by the relatively short time of follow-up that forbids any conclusions that the improvement could be sustained over a longer time period. However, it should be noted that patients with BPH eligible for surgical intervention usually remain on a waiting list for a limited period. Serum T was measured by an immunoassay rather than liquid chromatography-mass spectrometry; however, the measurement was made in the Careggi University Hospital laboratory, which performs a large number of measurements monthly for both clinical practice and research purposes and undergoes routine quality controls. In addition, it served as the central laboratory for immunoassay hormone assessment of the European Male Ageing Study showing a close agreement between T measured by immunoassay and liquid chromatography-mass spectrometry [46].

## Conclusions

This study is the first to provide experimental proof for the anti-inflammatory role of T on the prostate and thus to highlight its potential benefits in contraposition with the historical view of a detrimental effect. TTh in TD men with MetS and candidates for surgery for BPH improves prostatic inflammatory features thus ameliorating one of the pathogenic components of BPH. Decreased inflammation is not accompanied by a consistent improvement in urinary symptoms, which, although not supporting a role for T as an effective medicine for LUTS, reassures us on the safety of TTh even in subjects with BPH of surgical significance.

## Supplementary Information

Below is the link to the electronic supplementary material.

Supplementary file1 (PDF 352 KB)

Supplementary file2 (DOCX 14 KB)

## Data Availability

Some or all datasets generated and/or analyzed during the current study are not publicly available but are available from the corresponding author upon reasonable request.
