# Supplementary material for: Testosterone does not affect lower urinary tract symptoms while improving markers of prostatitis in men with benign prostatic hyperplasia: a randomized clinical trial
Source: J Endocrinol Invest. 2022 Mar 17;45(7):1413–25. doi: 10.1007/s40618-022-01776-9 (PMC9184417; doi:10.1007/s40618-022-01776-9)
Supplement: Supplementary file 1 — Supplementary file1 (PDF 352 KB) [file 40618_2022_1776_MOESM1_ESM.pdf]

Effectiveness of testosterone replacement therapy (TRT) on prostatic gland in hypogonadal patients affected by benign prostatic hyperplasia (BPH) and metabolic syndrome (MetS)

EUDRACT: 2012-003494-26

NCT02366975

Promoter Name Surname Prof. Mario Maggi

Address Viale Pieraccini 6, 50139 Florence

Tel 0554271415
Fax 0554271413
E-mail m.maggi @ dfc.unifi.it

Clinical center coordinator
Name SODC Sexual Medicine and Andrology, AOU Careggi, Florence, Italy

Principal Investigator

Prof. Mario Maggi

Investigators

SOD Sexual Medicine and Andrology, AOU Careggi: Dr. Linda Vignozzi; Dr. Giulia Rastrelli, Dr. Francesco Lotti

SOD Urology, AOU Careggi: Prof. Marco Carini

SOD Anatomic Pathology, AOU Careggi: Prof. Gabriella Nesi

Satellite clinics - Investigators of the major satellite clinics: none

Statement of the principal investigator of compliance with the Declaration of Helsinki, the national legislation and the Protocol

I, the undersigned Prof. MARIO MAGGI, declare to know the study protocol and that this is carried out as described and in accordance with:

- principles declared in the Declaration of Helsinki;

-Good Clinical Practice;
-EU directives (EU law 200/2001 and following) and national regulatory requirements of reference (Legislative Decree 211/2003, Legislative Decree 200/2007 and following)
Any changes to procedures will be made only to protect the safety, rights and welfare of those involved in the study.

I declare to coordinate the study by ensuring that all those who collaborate in its implementation will be aware of the protocol and any amendments and will act in full awareness of their obligations.

In witness
Date ______12.04.2012________________________________ Signature

Introduction

Benign prostatic hyperplasia (BPH) is one of the most common chronic diseases in men (50% in males between 50-60 years and 90% of males>80years; McVary KT, 2006), which is clinically manifested by the so called lower urinary tract symptoms (LUTS), generally referred to as BPH/LUTS, that have a major impact on quality of life in the elderly population. LUTS include irritative (urgency, nocturia, increased micturition frequency) and obstructive symptoms (hesitancy, intermittent urinary stream, sensation of incomplete emptying). Histologically, BPH can be defined as an increase in volume of the transactional (periurethral) area of the prostate gland associated with a tissue remodelling that involves both the epithelial component and the fibromuscular stroma (Fibbi et al., 2010).

Preclinical studies and clinical trials have demonstrated that the chronic inflammatory process of the prostate actually is the primary cause for the development of BPH/LUTS. In fact, an inflammatory infiltrate is associated with the release of cytokines and chemokines (such as interleukin-8; IL-8) capable, not only to induce the recruitment of other inflammatory cellular components, but also to stimulate the production of growth factors for the stromal cells of the prostate (Fibbi et al., 2010). An increase of IL-8 in the semen is considered a surrogate marker of prostate inflammation and BPH (Pen et al., 2007; Buckles et al., 2010).

Although aging is considered as the main risk factor for the development and progression of BPH, it has recently been shown that BPH/LUTS may reflect other systemic diseases (Moul & McVary, 2010). A strong and independent association between LUTS and metabolic syndrome (MetS), another high prevalent disease in aging population, has been reported (Mongiu & McVary, 2009; Moul & McVary, 2010; Gorbachinsky et al., 2010). According to the classification of the American Heart Association (AHA)/National Heart, Lung, and Blood Institute (NHLBI) (Alberti et al., 2009), the definition of MetS requires the presence of three or more of the following cardio-metabolic risk factors: visceral obesity (waist circumference; WC>102 cm), hypertension (blood pressure; BP≥130/85 mmHg or specific treatment), hypertriglyceridemia (triglycerides≥150 mg/dL or specific treatment), reduced levels of high-density lipoprotein (HDL)-cholesterol (≤40 mg/dL or specific treatment), fasting hyperglycemia (fasting glucose≥100 mg/dL or specific treatment). Recent studies have shown that MetS is associated not only with cardiovascular and metabolic diseases, but also with andrological disorders, such as sexual dysfunction, male infertility and BPH/LUTS. Therefore, MetS should be considered more properly as a condition characterized by a very heterogeneous clinical presentation (Gorbachinsky et al., 2010). Epidemiological studies have documented that patients with increased body mass index (BMI) suffer more frequently from LUTS than patients with normal BMI (Rohrmann et al., 2004; Seim et al., 2005). In a large scale population study, Laven et al. have shown that the presence of LUTS is associated with the presence of visceral obesity (Laven et al., 2008). Other factors of MetS, such as hypertension, type 2 diabetes mellitus (Michel et al., 2000; Michel et al., 2004; Tomita et al., 2009), hyperglycemia and low HDL-cholesterol (Martin et al., 2011) have been associated with LUTS. In agreement with these data, in the Boston Area Community Health Studies (BACH), a positive association between intake of calories (and especially that enriched in polyunsaturated fatty acids) and LUTS has been demonstrated (Litman et al., 2007).

Our research group has recently demonstrated a positive correlation between BMI and ultrasound and biochemical (such as increased concentrations of IL-8 in the semen) features typical of prostatic inflammation. In particular, a higher BMI was associated with the presence of ultrasound characteristics indicative of an inflammatory process (macrocalcifications, inhomogeneity, higher peak systolic velocity; PSV) and with higher values of IL-8 in the seminal fluid (Lotti et al., 2011).

Preclinical studies have also shown a close association between prostate inflammation and tissue remodelling associated with MetS. In particular, our group has recently developed an animal model of MetS that perfectly mimics the human phenotype. In fact, adult male rabbits fed with a diet enriched in fat (peanut oil and cholesterol) for 12 weeks, developed all the clinical features of MetS, including fasting hyperglycemia, hypetriglyceridemia, reduced levels of HDL-cholesterol, increase in BP and visceral obesity. In these animals, similarly to what happens in humans, we have also seen the development of a testosterone (T) deficiency (hypogonadotropic hypogonadism, Filippi et al., 2009; Vignozzi et al., 2012) and an inflammation of the prostate associated with tissue remodelling (Vignozzi et al., 2012). In fact, in the prostates of rabbits with MetS, we have shown the presence of an important inflammatory infiltrate with immunopositivity for the markers of leukocytes (T-cell surface glycoprotein cluster of differentiation 45; CD45), macrophages (monoclonal antibody RAM-11 and T-cell surface glycoprotein cluster of differentiation 68; CD68) and granulocytes (lactoferrin; LTF) which infiltrate both the stroma and the glandular epithelium. Histological analysis also highlighted an important fibrosis and a marked hypoxia (assessed with the specific marker of tissue hypoxia, Hypoxyprobe ®). The prostate of rabbits with MetS also showed a significant increase in all the non-specific inflammatory markers (cyclooxygenase-2; COX-2; receptor for advanced glycation endproducts; RAGE) and specific macrophages (toll-like receptor 2; TLR2; toll-like receptor 4; TLR4; six-transmembrane protein of prostate 2; STAMP2), granulocyte (LTF) and lymphocytes (T-cell surface glycoprotein

cluster of differentiation 4; CD4; T-cell surface glycoprotein cluster of differentiation 8; CD8; T-box transcription factor; T-bet; GATA binding protein 3; GATA3; retinoic acid receptor-related orphan receptor gamma (t); RORγt) markers as measured by quantitative gene expression analysis (quantitative real time reverse transcriptase-polymerase chain reaction; qRT-PCR). Even the markers of tissue remodelling and myofibroblastic activation (transforming growth factor; TGF; transgelin; SM22-α; α-smooth muscle actin; α-SMA; Ras homolog family member A; RhoA; Rho-associated coiled-coil containing protein Kinase 1; ROCK1; Rho-associated coiled-coil containing protein Kinase 2; ROCK2) are high, supporting the evidence of a marked histological tissue fibrosis (Vignozzi et al., 2012). Inflammation, fibrosis and hypoxia are considered pathological processes tightly interconnected with each other. In addition, their concomitant presence in the prostate gland characterizes the transition from prostatic inflammation to the development of BPH (Fibbi et al., 2010). Therefore, MetS appears to be closely related to the development of LUTS/BPH. A common factor between these two clinical conditions is represented by T deficiency, also called hypogonadism (Cohen 2009; Corona et al., 2009). It is well-known that hypogonadism may be considered as a typical clinical feature of MetS, and more recently an association between hypogonadism and LUTS/BPH has also been demonstrated. Some prospective (Kristal et al., 2008; Trifirò et al., 2010) and cross-sectional (Schatzl et al., 2000; Tan et al., 2003; Roberts et al., 2004; Miwa et al., 2008; St Sauver et al., 2011) studies have shown that there is an inverse association between serum T levels and the presence of LUTS/BPH. T replacement therapy (TTh) of hypogonadism in patients with MetS has been shown to improve many cardiometabolic parameters including: hyperglycemia, reduced glucose tolerance, visceral adiposity and hypertension (Corona et al., 2011). However, TTh is limited by concerns for its potential negative effect on prostate tissue, mainly because T is known as the hormone responsible for the development and growth of the prostate gland. On the contrary, some pilot studies have shown that TTh in hypogonadal (HG) subjects with LUTS/BPH and MetS (Haider et al., 2009) led to an improvement of LUTS.

In agreement with these clinical data, in our experimental model of MetS, treatment with T not only improved many parameters of MetS (such as hyperglycemia, visceral obesity, hypertension), but also completely prevented the development of inflammation in the prostate, reducing gene and protein expression of all studied inflammatory and fibrotic markers. These pre-clinical data indicate that TTh reduces the inflammation associated with prostatic MetS, thus representing a therapeutic option to reduce the risk of development and progression of BPH (Vignozzi et al., 2012).

Rationale

In literature there is a lot of evidence showing that MetS is associated with BPH/LUTS. There is also evidence that hypogonadism is associated with both conditions, thus being one of the most probable pathogenetic factor underlying the association between MetS and BPH/LUTS.

Preliminary evidence from observational clinical studies has shown that TTh in HG patients with MetS reduces LUTS associated with BPH. Preclinical studies performed by our research group showed in an experimental model of MetS the occurrence of marked inflammation and tissue remodelling of the prostate gland, which is prevented by TTh (Vignozzi et al., 2012). There is therefore a need for a clinical trial to demonstrate the effect of TTh in reducing the inflammation of the prostate and its effectiveness in improving the symptoms related to inflammation in patients with BPH associated with MetS and T deficiency.

Aim of the Study

The aim of the present study is to evaluate the effectiveness of TTh compared with placebo in reducing signs and symptoms of prostatic inflammation and LUTS in HG patients with MetS and BPH who are candidates for simple prostatectomy. For this purpose, clinical (assessment of specific symptoms of prostatitis through the National Institutes of Health-Chronic Prostatitis Symptom Index, NIH-CPSI questionnaire; assessment of LUTS using the International Prostate Symptom Score; IPSS), ultrasound (transrectal ultrasound; TRUS; evaluation of markers of prostatic inflammation: macrocalcifications, inhomogeneity etc.), urinary and histologic (histomorphometric and immunohistochemical analysis of prostate samples derived from patients enrolled in the study) assessments will be performed.

Objectives

<u>Primary objective:</u> evaluation, in HG patients with both BPH and MetS, of the effectiveness of 6-months TTh compared with placebo in improving symptoms of prostatitis (evaluated using the NIH-CPSI questionnaire) and LUTS (assessed using the IPSS questionnaire).

<u>Secondary objectives:</u> evaluation, in HG patients with both BPH and MetS, of the effectiveness of 6-months TTh compared with placebo in improving:

- ultrasound characteristics typical of the inflammatory process in the prostate (e.g., presence of macrocalcifications, increase in the volume of the prostate gland);
- prostatic inflammation, assessed by immunohistological analysis and gene expression of inflammatory markers on prostate tissue obtained from surgical specimens of prostatectomy;
- metabolic parameters (glucose, insulin, total cholesterol, HDL-cholesterol, triglycerides, glycated hemoglobin; HbA1c, BP, WC, BMI).

Study Population

Clinical setting: outpatients with BPH and MetS in waiting list for surgery.
Definition of the population eligible for the study: adult men with BPH in waiting list for surgery and that met the AHA and the NHLBI diagnostic criteria for MetS (Alberti et al., 2009) will be considered eligible for the study.

Inclusion criteria:

1. Male subjects aged≥18 years in waiting list for simple prostatectomy for BPH;
2. Diagnosis of MetS according to the AHA/NHLBI criteria (see below);
3. Presence of moderate prostatitis-like symptoms, as defined by an overall score on the NIH-CPSI>15 (Propert et al., 2002);
4. Capacity to give consent for study participation, after being adequately informed of the aims, benefits, risks, time and motion of the study.

Exclusion criteria:

1. Participation in another clinical trial;
2. Previous diagnosis, presence or suspected prostate or breast cancer;
3. Prostate specific antigen (PSA) values>10 ng/mL;
4. Hematocrit≥52%;
5. Use of 5α-reductase inhibitors in the previous three months;
6. Presence of a serious organic or mental disease or other conditions that may affect the compliance to the study;
7. Presence of severe allergy or hypersensitivity to the study drug (active ingredient or excipients of the formulation).

Study Design and Protocol

The Florence PROTEST is designed as a double-blind placebo-controlled randomized clinical trial (RCT) that will be conducted at a single center at the Careggi Hospital - University of Florence. The study aims at enrolling 120 men. The patients' screening will be conducted at the Urology Unit and the Sexual Medicine and Andrology Unit. The recruitment will take place at the latter Unit. After signing the informed consent, all patients will undergo the baseline visit procedures (time 0 = V1). These will include the collection of fasting blood samples within 11 a.m. NIH-CPSI (Litwin et al., 1999) and the IPSS (Barry et al., 1992) will be administered for the assessment of prostatitis-like symptoms and LUTS, respectively. For each patient, height, weight, WC and BP will be measured and patients will undergo a prostatic TRUS with color Doppler method (Lotti et al., 2010, 2011).
Total T and sex hormone binding globulin (SHBG) will be measured and, according to total and free T, patients will be classified into eugonadal (total T≥12 nmol/L and free T≥225 pmol/L) or HG (total T<12 nmol/L and/or free T<225 pmol/L) (Wang et al., 2009). As soon as the hormone results become available (usually 2-3 days), HG men will be randomized to receive T gel 2% (Tostrex®, Kyowa Kirin S.r.l.) or placebo 5 g daily for 24 weeks. The duration of treatment corresponds to the minimum waiting time for surgery for simple prostatectomy for BPH in the Urology Unit. T or placebo gel will be provided to patients together with accurate instructions on how to use it according to the Kiowa Kirin's datasheet. The use of placebo is considered free from possible harm to the patient because of the fact that not all HG patients in clinical practice receive hormonal replacement therapy and that the conditions associated with hypogonadism leading to increased morbidity or mortality (e.g., osteopenia and osteoporosis, reduced muscle strength and altered glycolipid profile) have a multifactorial pathogenesis and a period of 6 months is not enough to get a significant worsening of these conditions. Substitution treatment is indeed very personal and only recommended in HG patients complaining of specific symptoms of androgen deficiency, such as sexual desire disorder, loss of muscle mass, depression, and therefore not for the simple detection of low levels of serum T (Bhasin et al., 2010). Enrollment, in fact, will occur among patients who will arrive at the observation of the Urology specialist for LUTS associated with BPH and in need of surgical treatment of simple prostatectomy.
After receiving the amount of study medication necessary to cover the entire study period, patients will be seen after 24 weeks (time +24 weeks= V2). Over this period, patients will receive periodic telephone contacts to

monitor medication compliance and possible adverse events (AEs). At V2, patients will undergo the same procedures as at V1. After this visit, patients will be admitted at the Urology Unit within the following weeks to undergo planned surgery for BPH. At surgery, samples of prostatic tissue will be harvested for molecular assessment. As for routine procedure, most of the removed tissue will be sent at the Anatomic Pathology Unit of our University Hospital for histopathological assessment. Besides the routine evaluation, the Inflammatory Score (IS) will be calculated (Nickel et al., 2001).
Before starting any study procedure, patients will be provided by study investigators with the relevant information, including rationale, objectives, time and efforts required from participants, possible benefits and risks. An informative sheet will be also provided for themselves and their general practitioner. If willing to participate, patients will be required to express their written consent by signing the informed consent form.

Study Procedures

More in detail:

1. Recruitment and informed consent: patients will be screened at the Urology Clinic directed by Prof. M. Carini at the Careggi Hospital – University of Florence, and informed by the physician who will evaluate the eligibility. If eligible, the patient's recruitment will take place at the Sexual Medicine and Andrology Unit at Careggi Hospital. After information is delivered to the patient, the informed consent for the study and a letter for the general practitioner will be given to him. If the patient agrees to be included in the study, he will sign the informed consent in front of the physician who proposed the study.
2. Clinical procedures: 2 medical visits (V1 and V2), 2 prostate TRUS (V1 and V2), 2 blood samples (V1 and V2), administration of questionnaires including NIH-CPSI, IPSS, Ageing Male Scale (AMS) and International Index of Erectile Function-5 (IIEF-5) (V1 and V2) and structured interview ANDROTEST (V1).

The patient will be given a telephone number where he can contact the study physician.

The study is constructed as follows:

- At V1 (time 0): all patients enrolled will undergo a physical examination (weight, height, WC, BP) and will be provided a blood sample for the determination of the following indexed hormones (T, SHBG), metabolic parameters (total cholesterol, HbA1c), prostatic markers (PSA) and hematocrit. The following questionnaires will be administered: NIH-CPSI, to assess the presence and severity of prostatitis; the IPSS, to evaluate LUTS, the AMS and ANDROTEST to evaluate androgen deficiency symptoms, and the IIEF-5 to assess erectile function. All questionnaires will be filled out by the patient. The calculation of the relative scores will be carried out by the examiner only at the end of the study in order to maintain blind the procedures. A TRUS with color- Doppler method will be also performed after blood samples are collected (Lotti et al., 2010 and 2011).

As soon as the results of hormones measured at V1 visit are available, patients will be divided into HG and eugonadal based on the levels of total T and/or free T (calculated using the Vermeulen formula; Vermeulen et al., 1999). HG patients will be randomized into two distinct treatment groups: placebo or 2% T gel (5 g/die).

- At time 24 visit: all patients will be re-evaluated by physical examination (weight, height, WC, BP) and a blood sample will be provided for the determination of the following indexed hormones (T, estradiol, SHBG), metabolic parameters (glucose, insulin, total cholesterol, HDL-cholesterol, triglycerides, HbA1c), prostatic markers (PSA) and hematocrit. All the questionnaires administered at baseline visit (NIH- CPSI to evaluate changes in the severity of prostate inflammation, IPSS to evaluate changes in the degree of LUTS) will be re-assessed at time 24 visit. A TRUS with color-Doppler method (Lotti et al., 2010 and 2011) will be also performed.

After time 24 visit, the patient will undergo simple prostatectomy for BPH. Prostate samples will be used for routine histological examinations. A part of the sample will be used for immunohistochemical analysis of inflammatory tissue components and for the analysis of gene expression of inflammatory markers and myofibroblastic activation (see below).

MetS Diagnosis
MetS will be diagnosed according to the AHA/NHLBI criteria (Alberti et al., 2009). WC will be measured in standing position, using a tape measure rested on the skin at the mid-point between the iliac crest and the lower rib margin. Patients will be asked to relax with arms on their sides and to breathe in and out, looking straight ahead. The measure will be repeated thrice and the mean value will be registered using the nearest 0.1 cm. Fasting levels of HDL-cholesterol, triglycerides and glucose will be retrieved from available patients' medical records taking into account the last value available that should not precede the screening evaluation of 3 months. BP will be measured

in the sitting position, after at least 5 minutes of rest. Systolic and diastolic BP will be measured twice 5 minutes apart and the mean value will be registered. Patients will be asked to report any medication used and those relevant for MetS criteria will be considered for the diagnosis.

Blood Measurements
Total T, SHBG and PSA will be measured by established commercial platform immunoassays (Modular E170 platform electrochemiluminescence immunoassays-Roche Diagnostics-Mannheim, Germany). These measurements will be performed at the central laboratory of our University Hospital together with the assessment of hematocrit. Free T will be calculated (cFT) according to the Vermeulen's formula (Vermeulen et al., 1999).

Prostatic Transrectal Ultrasound
The prostate will be studied using the console MyLab Class C (Esaote SpA, Genova, Italy), scanning the organs at 5 mm intervals as previously described (Lotti et al., 2010 and 2011), using a transrectal biplanar probe (linear transducer U533L 7.5 MHz; convex transducer U533C 6.5 MHz). To prevent bias on the part of the examiner, TRUS will be performed intermittently by two experienced physicians (F.L. and S.C.) who will be unaware of the clinical data. Prostate volume will be calculated using the planimetric method, measuring the three diameters and using the ellipsoid mathematical formula (Lotti et al., 2010 and2011). Prostate color Doppler ultrasound features will be defined as reported previously (Lotti et al., 2010 and 2011).

Surgical Procedures and Collection of Prostate Specimens
Simple prostatectomy, namely transvesical prostatectomy or transurethral resection of the prostate (TURP) will be performed, with resectoscopes and cutting loops, removing the hyperplastic prostatic tissue of the transition zone. Transvesical prostatectomy or TURP will be performed according to the surgeon decision. The quality of samples collected from TURP or transvesical prostatectomy is comparable. Surgical specimens will be collected with sterile procedure. All samples will be obtained from at least three different sites of the adenomatous tissue.

RNA Extraction and Quantitative RT-PCR Analysis
Isolation of total RNA from prostate specimens will be performed using TRIzol reagent (Life Technologies, Paisley, UK) and Qiagen RNeasy Mini Kit (Qiagen, Hilden, Germany), both following the manufacturer's instruction. cDNA synthesis will be carried out using the iScript™ cDNA Synthesis Kit (Bio-Rad Laboratories, Hercules, CA) and qRT-PCR amplification and detection will be carried out using SsoAdvanced™ Universal SYBR® Green Supermix on a CFX-96 Thermal Cycler PCR Detection System (both Bio-Rad Laboratories), as previously described (Vignozzi et al., 2012). Specific PCR primers for human target genes were designed on sequences available at NCBI GenBank (http://www.ncbi.nlm.nih.gov) or Ensemble Genome (http://www.ensembl.org). The 18S ribosomal RNA subunit will be used as the reference gene for the relative quantization of the target genes based on the comparative threshold cycle (Ct) $2^{-\Delta\Delta Ct}$ method (Livak & Schmittgen, 2001).

Pathological Characterization of Prostatic Inflammatory Infiltrates
Prostatic samples will be evaluated for the presence of an inflammatory infiltrate, according to the standardized classification system of chronic prostatitis of the National Institutes of Health (Nickel et al., 2001). All samples will be double-checked by two independent pathologists, blinded to clinical findings that assessed all the available glass slides. The number of glass slides per patient will vary depending upon the amount of resected prostatic tissue. In particular, the following characteristics of infiltrate will be evaluated and scored: prevalent anatomical location (1=stromal; 2=periglandular; 3=glandular), grade (1=mild; 2=moderate; 3=severe) and extent (1=focal; 2=multifocal; 3=diffuse). For statistical analysis purpose, the IS will calculated as the sum of the three different histological inflammatory parameters, as previously described.

Endpoints

Primary endpoint: improvement, over 24 weeks, of prostatitis symptoms and LUTS in HG men with MetS and BPH treated with TTh compared with the placebo group. Improvement will be defined by at least two of the following: (i) reduction of at least 2 points of the NIH-CPSI score, (ii) reduction of at least 3 points of "total IPSS" score, and (iii) reduction of at least 1 point in the "IPSS bother" score. In conclusion, the "responders" will be those patients who show a significant improvement of at least two of the three questionnaires listed above, namely: NIH-CPSI, "total IPSS" and "IPSS bother". Subjects that will not provide questionnaires at V2 will be treated as "non responders". The primary analysis will be based on the intention-to-treat (ITT) population, namely all patients that will be randomized to either of the treatment arms.

The NIH-CPSI questionnaire is a validated scoring system, even in its Italian version (Giubilei et al., 2005), to evaluate three distinct domains: pain, urinary symptoms and impact on quality of life of an inflamed prostate. Patient's symptoms are assessed for a total of 43 points (21 pain, 10 urinary symptoms and 12 impact on quality of life). The questionnaire will be filled out directly by the patient but the score will be calculated by the medical examiner. According to the score of this questionnaire, the severity of prostatic inflammation may be divided into mild (0-14 points), moderate (15-29) or severe (> 30).
The IPSS questionnaire has been recommended for initial assessment of the subjective severity of LUTS/BPH. IPSS evaluates different components of LUTS: obstructive and irritative symptoms ("total IPSS": 0-35) and the quality of life ("bother IPSS": 0-5).

Secondary endpoints:

- statistically significant reduction of prostate volume in TTh group vs. placebo group measured by TRUS examination at both visits. Prostate volume will be measured using the method as described by planimetric (Behre et al., 1995 and Vicari, 1999);
- statistically significant reduction in the number and diameter of intraprostatic macrocalcifications in the TTh group vs. placebo group measured by TRUS examination at both visits. Macrocalcifications will be defined as calcification of diameter>3 mm, as already reported by Dahnert et al., 1986, Dagash & Mackinnon, 2007, Isidori & Lenzi, 2008 and Lotti et al., 2011.
- statistically significant reduction of inflammation in prostatic tissue in in the TTh group vs. placebo and vs. eugonadal, as assessed by:

• histological analysis. The degree of inflammation of the prostate will be defined through the use of a score based on the NIH-CPSI score (Nickel et al., 2001). The parameters of the score will be evaluated by optical microscopy of tissue obtained from the piece at the Anatomic Pathology Unit of Careggi Hospital;

• gene expression analysis of inflammatory markers in the prostate tissue. The expression of the following genes, involved in the prostatic inflammatory process, will be analyzed: TLR2, TLR4, CD4, CD8, LTF, STAMP2, RAGE, COX-2, IL-1β, IL-4, IL-6, IL-8, IL-10, IL-12A, IL-17, oxidized low-density lipoprotein receptor-1 (LOX-1), insulin receptor substrate 1 (IRS1), monocyte chemoattractant protein-1 (MCP-1), Interferon gamma-induced protein-10 (IP-10), TNFα (tumor necrosis factor α), RhoA, ROCK1, ROCK2, AR (androgen receptor), estrogen receptor α and β (ERα , ERβ), G protein-coupled receptor 30/G protein-coupled estrogen receptor 1 (GPR30/GPER1), progesterone receptor (PR) , ROR γt, GATA3, PSA and T-box transcription factor 21 (TBX21);

- significant reduction in the prevalence of MetS in the study population in the TTh group vs. placebo;
- improvement in sexual function assessed by the IIEF-5 questionnaire in the TTh group vs. placebo.

Endpoint of exploratory objectives: statistically significant reduction in the number of patients requiring surgical treatment for BPH in the TTh group vs. placebo.

Registration Procedures

To be included in the study, each patient will be required to sign an informed consent form, specifically for this study. All procedures useful for the verification of eligibility must be completed before the formal registration of patients in the study. On the contrary, any therapeutic procedure provided by the experimental protocol can be implemented only after the completion of the formal registration of patients in the study. To apply for the enrollment of a new patient in the study, registration procedures will be performed through the web-based system and clinicaltrials, administered by the Coordinating Center for Clinical TrialsITT of Careggi Hospital, University of Florence. The address of the site's home page, from which procedures can be used is: www.eclintrials.org/ect. Patients will be centrally randomized 1:1 using an interactive web response system (IWRS) and the minimization algorithm. Randomization will be stratified by age < or ≥45 years. Once classified as HG, a randomization number will be assigned to patients, which corresponds to a set of gel packages for covering the entire study period. T gel and placebo present as indistinguishable packages identified only by a number corresponding to the random number assigned to each patient. The request for a new patient enrollment will be made by completing the registration form contained in the case report form (CRF) of the study. This form will contain all the information related to the selection criteria. Upon registration, the system will inform the investigator and the code number of the patient who will later be used on all documentation produced during the study, including CRFs. Before the registration request is submitted, it is the responsibility of the investigator to ensure that the patient meets all eligibility criteria provided by the study. Any patient, at any time, even after the recording, may withdraw voluntarily from the clinical study.
Patients, study personnel performing visit procedures, and personnel involved in laboratory procedures or data

analysis will be all blinded to the treatment arm. In addition, personnel performing laboratory analysis on prostate tissue will be blinded to clinical data.

Statistical Analysis

The study is designed to have an 80% power at an alpha level of 0.05, with the use of two-tailed chi-square statistical test to detect a difference in the rate of improvement between the HG groups of 25%. These calculations are based on a sample of 38 participants per HG arm. Since we are interested in recruiting also eugonadal men as comparison group for the analyses on the prostate samples, we plan to recruit overall 120 men with MetS and BPH in order to achieve 76 HG men. The proportion of HG subjects who will improve urinary symptoms will be assessed by the odds ratio (OR) and the comparison between the improvement rates in the two groups will be evaluated with the chi-square test, using the HG+placebo arm as the comparator. For the secondary outcomes, the distribution of the continuous variables will be assessed. Normally distributed variables will be expressed as mean±standard deviation, whereas non-normally distributed variables will be expressed as median [interquartile range]. The change over time in the two treatment groups will be assessed by the multilevel mixed-effects linear regression using the treatment arm, the time points and their interaction as independent variables, the measure of the variable at time 24 as the dependent variable and the patients' ID as random effect. The analyses will be adjusted for relevant confounders, if needed. For each outcome, the estimated marginal mean (EMM) and 95% confidence interval (CI) for each HG arm will be reported with the p-value within group (p_{within}). In addition, the contrast of EMM between the HG groups at time 24 and 95% CI with the p-value between groups ($p_{between}$) will be reported. For measures on prostate samples, all the three study arms will be considered. The differences between the three groups for non-normally distributed parameters will be assessed by unpaired two-tailed Kruskal-Wallis test, followed by Mann-Whitney two sample statistic, for normally distributed variables by one-way ANOVA test with post-hoc analysis (Fisher's Least Significant Difference). All reported p values will be two-sided and values below 0.05 will be considered statistically significant. All statistical analyses will be conducted using Stata MP 13.1 for Windows (StataCorp, College Station, TX, USA).

Management of Adverse Events

AEs will be reported in conformity with the national regulatory framework (Legislative Decree 211/2003).

<u>Adverse Events/Adverse Drug Reactions</u>
"Adverse event" means any untoward medical event occurring in a patient or a person involved in a clinical trial, which was given a medicine, and does not necessarily have a causal relationship with this treatment. An AE can therefore be any sign (including an abnormal laboratory finding), unfavorable or unintended symptom or disease associated with the use of the drug product (investigational) for the coincidence in time, whether related or not to the medicinal product (investigational) ("Notes for Guidance for Good Clinical Practice" CPMP/ICH/135/95).
Any person who sustains an AE will be examined by a doctor as soon as possible. The doctor will do what is necessary for the safety and welfare of the subject. All anomalies will be followed until healing or clinical stabilization. The AE is described in the medical records using standard medical terminology (MedDRA®) to avoid the use of vague, ambiguous or colloquial expressions. The investigator will evaluate all AEs for severity and relationship to the investigational product and return the result and what action to take.
"Adverse drug reaction" (ADR) means any untoward and unintended reaction to a drug being tested, regardless of the dose administered. All noxious and unintended responses to a medicinal product related to any dose should be considered as an ADR.
"Responses to a medicinal product" means that there is at least a reasonable possibility of a causal relationship between a medicinal product and an AE, namely, that the relationship cannot be excluded. ("Notes for Guidance for Good Clinical Practice" CPMP / ICH/135/95).

Serious Adverse Events/Serious Adverse Reactions

A serious adverse event (SAE)/serious adverse reaction (SADR) is "any AE or adverse reaction, at any dose, that:

- succeeds in death;
- endangers the life of the subject;
- requires hospitalization or prolongs a hospital stay;
- causes serious or prolonged disability or incapacity;
- involves a congenital anomaly or birth defect. "

("Note for Guidance for Good Clinical Practice "CPMP/ICH/135/95).
An AE/ADR is a non-serious AE/ADR that does not meet the above described criteria.

Unexpected Adverse Reactions
An unexpected adverse reaction is an "adverse reaction in nature or severity that is not predictable based on the product information (such as those shown in the investigator's brochure, if the product is under investigation or, in the case of an authorized product, in the summary of product characteristics). (“Note for Guidance for Good Clinical Practice" CPMP/ICH/135/95).

Adverse Events/Serious Adverse Reactions
The investigator shall report immediately to the promoter, but not later than 5 calendar days, any AE or serious problem occurring during the study, filling in the space provided in the confidential medical records. This report will be followed by a detailed written report to be sent to the promoter within 3 calendar days after the first report. All fatal or life-threatening AEs must be reported to the initiator by sending the "Urgent Report Form" immediately and not later than 24 hours from when it came to the experimenter knowledge. Within 48 hours, this report will be followed by a complete detailed report on the case.
The sponsor will ensure that all relevant information about suspected serious or unexpected adverse reactions (fatal or potentially fatal), are recorded and notified as soon as possible to the Ministry of Health, as well as to the Board for ethical questions, and in any case within 7 calendar days from when the sponsor is aware of the case, and that further relevant information is communicated within 8 days after the first report.
Any serious AE must be notified to the promoter by fax to the following contact: Dr. Linda Vignozzi: 0554271413 or 0554271371.
All other suspected SADR (non-fatal or potentially fatal) are reported to the Ministry of Health and the Committee for ethical questions, as soon as possible and no later than 15 days from the date on which the promoter became aware for the first time.
Once a year throughout the clinical trial, the sponsor must provide the Ministry of Health and the involved Ethics Committees a list of all suspected SADR observed during this period and a report on security for persons subjected to clinical trials.

Non-serious Adverse Events
The investigator is obliged to report all AEs to the sponsor, even if it is not serious. This information will be collected in medical records.

Instructions for the Office Staff about Unusual or Severe Signs or Symptoms
Each subject will be told who to contact in case of occurrence of any unusual or severe symptom or sign after treatment. Individuals who will experience severe systemic reactions, if possible, should be visited in the institution at the time of maximum expression of symptoms and will be followed clinically until resolution.
Whatever causes the withdrawal of the subject from the study, it will be reported in the appropriate section of the medical record. All AEs will be followed until the healing and/or diagnosis. If an incident is not resolved at the conclusion of the study, the investigator will evaluate whether to allow the follow-up. All drugs taken during the study to treat AEs or previous diagnosed diseases will be recorded in medical records.
In the case it is possible to formulate a diagnosis, it is preferable to give the diagnosis rather than using a series of terms related to it. When a syndrome is reported, the associated signs and symptoms should be identified as part of the syndrome and not as separate events.

Causation
The investigator will evaluate the association between AEs and treatment according to the following definitions:

Some
A clinical event - including abnormality of laboratory tests - that follows, with a reasonable time sequence, the administration of the drug but that cannot be explained by a concurrent illness or by other drugs. The reaction must have already been observed for the suspected drug. The reaction should improve with "dechallenge" and reappear with “rechallenge."

Likely
A clinical event - including laboratory test abnormality - which follows, with a reasonable timeline, the administration of the drug but that could not be explained by a concurrent disease or other drugs, and whose response to "dechallenge" is clinically acceptable. The data of "rechallenge" are not required.

Possible
A clinical event - including laboratory test abnormality - which follows, with a reasonable timeline, the

administration of the drug but that may also be explained by a concurrent disease or other drugs. Information relating to the suspension of the drug may be missing or uncertain.

Unlikely

A clinical event - including laboratory test abnormality - whose temporal sequence of drug administration makes the causal relationship improbable, and where other drugs or underlying diseases provide plausible explanations.

Not Classified

A clinical event for which there is insufficient information at the time of the investigation and for which more data are needed for a proper assessment.

Not Classifiable

A clinical event for which the information is inadequate and/or inconsistent and does not allow a reasonable assessment.

Abnormalities in laboratory values

The investigator must evaluate the clinical significance of all abnormal laboratory values according to the definition of standard reference laboratories. Any clinically significant abnormality should be fully investigated. “Clinically significant" means any fault, according to the investigator, that is an important clinical problem requiring medical attention or that otherwise meets the definition of a SAE. When clinically indicated, further analysis or evaluation should be performed to determine the significance or the etiology of an abnormal result or to monitor the course of an AE. Any persistent abnormal value must be followed at the discretion of the investigator. Clinically significant abnormal results will be documented in the appropriate form of medical records.

Comorbidities and underlying diseases

Comorbidities (including signs/symptoms of a preexisting medical condition) that are present during or prior to administration of the investigational product and which occur with the same severity, frequency or duration of administration of the study drug, must be reported in appropriate form of medical records. However, cases that show an increase in severity or duration of pre-existing or concurrent illness should be reported as AEs.

Ethical aspects and respect of confidentiality

The experimenters ensure that the study will be conducted in full conformance with the international regulations [Dir EU 2001/20/EC] and its national transposition [DM July 15, 1997, Legislative Decree 211/2003, Legislative Decree 200/2007] on the clinical trial and the principles of the Declaration of Helsinki in order to ensure maximum protection of those involved. The principal investigator is committed to conducting the trial in accordance to what is written in this protocol and the Good Clinical Practice (GCP). The promoter of the study is committed to the protection of sensitive personal data, clinical or otherwise, of those involved in the study as defined in national legislation [D.Lvo. 196/2003].

Informed Consent

It is the responsibility of the investigators, or persons acting on their behalf, to obtain the informed consent of patients after adequate information about the aims, methods, anticipated benefits and foreseeable risks of the study. Investigators and those responsible should also inform participants that the non-participation or withdrawal of the same will not cause injury or damage to them.

Ethics Committee and Competent Authority

The promoter will provide reference to the Ethics Committee and the Competent Authority (General Director of Careggi Hospital) of the study protocol and all related documents provided to the patient (note and informed consent form). The approval of the Ethics Committee and Competent Authority must be obtained before any study-related procedure and must be documented through official communication to the investigator. If during the trial changes to the study protocol might be necessary, the sponsor shall submit to the Ethics Committee appropriate reference request for an amendment to the protocol, whose approval will follow the procedures established by the regulation of the same Ethics Committee.

Ownership of Data

The properties of the data, being independent study under the DM December 17, 2004, belongs to the promoter of the firm (DM 17 December 2004, Article 1, paragraph 2, letter c).

Final Report and Publication of Results

According to ICH-GCP, the head of the firm is committed to produce a report on the study, publish all the data collected as described in the protocol and to ensure that data are reported consistently and responsibly. In particular, the publication of data that result from this study will take place regardless of the results obtained. The transmission or dissemination of data, by means of scientific publications and/or presentation at conferences, meetings and seminars, participation in multicenter studies, will be used only as a result of purely statistical processing of the same, or otherwise completely anonymous. Responsible for the entire research and data processing is Prof. Mario Maggi, who led the study.

Independence of the Study
The firm has all the necessary requirements according to the DM December 17, 2004 (Art. 1, Section 1 and 2) for the definition of "clinical trial designed to improve clinical practice as an integral part of health care and not for industrial purposes."

Contributions and Conflict of Interest of the Head/s of the Study
Prof. Mario Maggi
Prof. Mario Maggi and Dr. Linda Vignozzi have designed this protocol. Dr. Linda Vignozzi wrote this Protocol.

Sources of Contributions
This trial was conceived independently of any commercial organization and will be coordinated, managed and analyzed in an independent form. Kiowa Kirin will provide Tostrex and placebo without any cost but will have no part in the study design, data analysis and interpretation or manuscript draft. The funds needed to support the additional costs involved for the conduction of the study are of membership units of Sexual Medicine and Andrology Unit, Department of Clinical Pathophysiology, University of Florence.

Insurance Coverage
Insurance coverage for patients and medical staff and nurses involved in the study, as determined by the DM December 17, 2004 (Article 2, Section 4), is indirectly falling within the insurance coverage provided for the clinical activity within Careggi Hospital (ad hoc stipulation insurance brokers winner of a tender procedure ESTAV 2010).

Data Collection
For the purposes of data collection procedures of the study, remote data entry will be available through the web based system and clinicaltrials, administered by the Coordinating Center for Clinical Trials ITT of Careggi Hospital, University of Florence. The address of the site's home page, from which procedures can be used is: www.eclintrials.org/etc.
The investigator will ensure that all data provided in each phase of the study are reported promptly on electronic CRFs, as instructed to do so. Automatic control of texture and consistency of data does not allow the transfer of incomplete or incorrect data. All corrections made on electronic CRFs will be stored by the system.
The system will track through the authentication data communicated at the time of access, the name of the user who made the data changes (inserts, updates), as well as date and time at which the transactions were made.
The investigator will be responsible for the preservation of the identification codes of each patient/subject (hospital code/local health unit identification code of the study/trial registration number) for a period of at least 7 years after completion or discontinuation of the clinical study.
Other original documents such as patient files and medical records must be kept for a maximum period of time allowed by the hospital, institution or private clinic, provided that it is not less than 7 years after completion or discontinuation of the study.
The investigators should also keep their own copies of CRFs and other trial-related documents for the same period of time.
In the event that the principal investigator should be transferred or retiring or declining to exercise its responsibility for the preservation of the records of documents related to the study in a 7-year period as specified above, this one will be required to transfer to the promoter, so that the documentation can be stored in sealed containers in the archives of the promoter. The inventory of stored documents will be kept by the investigator and a copy of it by the promoter.

Bibliography

Alberti KGMM, Eckel RH, Grundy SM, Zimmet PZ, Cleeman JI, Donato KA, et al. Harmonizing the metabolic syndrome: A joint interim statement of the international diabetes federation task force on epidemiology and

prevention; National heart, lung, and blood institute; American heart association; World heart federation; International atherosclerosis society; And international association for the study of obesity. Vol. 120, Circulation. Circulation; 2009. p. 1640–5.

Barry MJ, Fowler FJ, O'Leary MP, Bruskewitz RC, Holtgrewe HL, Mebust WK, et al. The American Urological Association symptom index for benign prostatic hyperplasia. J Urol. 1992;148(5 I):1549–57.

Bhasin S, Cunningham GR, Hayes FJ, Matsumoto AM, Snyder PJ, Swerdloff RS, Montori VM; Task Force, Endocrine Society. Testosterone therapy in men with androgen deficiency syndromes: an Endocrine Society clinical practice guideline. J Clin Endocrinol Metab. 2010;95(6):2536-59.

Cohen PG. Benign prostatic hyperplasia: the hypogonadal-obesity-prostate connection. Med Hypotheses. 2009 Aug;73(2):142-3.

Corona G, Mannucci E, Forti G, Maggi M. Hypogonadism, ED, metabolic syndrome and obesity: a pathological link supporting cardiovascular diseases. Int J Androl. 2009;32:587-98.

Corona G, Monami M, Aversa A, Tishova Y, Saad F, Lenzi A, Forti G, Mannucci E, Maggi M. Testosterone and metabolic syndrome: a meta-analysis study. J Sex Med. 2011 Jan;8(1):272-83.

Fibbi B, Penna G, Morelli A, Adorini L, Maggi M. Chronic inflammation in the pathogenesis of benign prostatic hyperplasia. Int J Androl. 2010 Jun 1;33(3):475-88.

Filippi S, Vignozzi L, Morelli A, Chavalmane AK, Sarchielli E, Fibbi B, Saad F, Sandner P, Ruggiano P, Vannelli GB, Mannucci E, Maggi M. Testosterone partially ameliorates metabolic profile and erectile responsiveness to PDE5 inhibitors in an animal model of metabolic syndrome. J Sex Med. 2009; 6:3274-3288.

Giubilei G, Mondaini N, Crisci A, Raugei A, Lombardi G, Travaglini F, Del Popolo G, Bartoletti R. The Italian version of the National Institutes of Health Chronic Prostatitis Symptom Index. Eur Urol. 2005Jun;47(6):805-11.

Gorbachinsky I, Akpinar H, Assimos DG. Metabolic syndrome and urologic diseases. Rev Urol. 2010;12:e157-80

Haider A, Gooren LJ, Padungtod P, Saad F. Concurrent improvement of the metabolic syndrome and lower urinary tract symptoms upon normalization of plasma testosterone levels in hypogonadal elderly men. Andrologia. 2009;41:7-13.

Krieger JN, Nyberg L and Nickel JC, NIH consensus definition and classification of prostatitis. JAMA. 1999;282:236–237.

Kristal AR, Schenk JM, Song Y, Arnold KB, Neuhouser ML, Goodman PJ, Lin DW, Stanczyk FZ & Thompson IM. Serum steroid and sex hormone-binding globulin concentrations and the risk of incident benign prostatic hyperplasia: results from the prostate cancer prevention trial. Am J Epidemiol. 2008;168:1416-1424.

Laven BA, Orsini N, Andersson SO, Johansson JE, Gerber GS, Wolk A. Birth weight, abdominal obesity and the risk of lower urinary tract symptoms in a population based study of Swedish men. J Urol. 2008;179:1891-1895.

Litman HJ, Steers WD, Wei JT, Kupelian V, Link CL, McKinlay JB; Boston Area Community Health Survey Investigators. Relationship of lifestyle and clinical factors to lower urinary tract symptoms: results from Boston Area Community Health survey. Urology. 2007;70:916-921.

Litwin MS, McNaughton-Collins M, Fowler FJ, Nickel JC, Calhoun EA, Pontari MA, et al. The National Institutes of Health chronic prostatitis symptom index: development and validation of a new outcome measure. J Urol. 1999;162(2):369–75.

Liwak KJ, Schmittgen TD. Analysis of relative gene expression data using real-time quantitative PCR and the 2(-Delta Delta C(T)) Method. Methods. 2001 Dec;25(4):402-8.

Lotti F, Corona G, Mancini M, Filimberti E, Degli Innocenti S, Colpi GM, Baldi E, Noci I, Forti G, Adorini L, Maggi M. Ultrasonographic and clinical correlates of seminal plasma interleukin-8 levels in patients attending an andrology clinic for infertility. Int J Androl. 2010 Oct 24. doi: 10.1111/j.1365-2605.2010.01121.x.

Lotti F, Corona G, Colpi GM, Filimberti E, Degli Innocenti S, Mancini M, Baldi E, Noci I, Forti G, Adorini L, Maggi M. Elevated body mass index correlates with higher seminal plasma interleukin-8 levels and ultrasonographic abnormalities of the prostate in men attending an andrology clinic for infertility. J Endocrinol Invest. 2011 Jul 7. [Epub ahead of print]

Martin SA, Haren MT, Marshall VR, Lange K, Wittert GA; Members of the Florey Adelaide Male Ageing Study. Prevalence and factors associated with uncomplicated storage and voiding lower urinary symptoms in community-dwelling Australian men. World J Urol. 2011;29:179-184.

McVary KT. BPH: epidemiology and comorbidities. Am J Manag Care. 2006;12,S122-S128.

Michel MC, Heemann U, Schumacher H, Mehlburger L, Goepel M. Effect of diabetes on lower urinary tract symptoms in patients with benign prostatic hyperplasia. J Urol. 2000;163:1725-1729.

Michel MC, Heemann U, Schumacher H, Mehlburger L, Goepel M. Association of hypertension with symptoms of benign prostatic hyperplasia J Urol. 2004;172:1390-1393.

Miwa Y, Kaneda T & Yokoyama O. Association between lower urinary tract symptoms and serum levels of sex hormones in men. Urology. 2008,72:552-555.

Mongiu AK, McVary KT. Lower urinary tract symptoms, benign prostatic hyperplasia, and obesity. Curr Urol Rep. 2009;10:247-253.

Moul S, McVAry KT. Lower urinary tract symptoms, obesity and the metabolic syndrome. Curr Opin Urol. 2010;20:7-12.

Nickel JC, True LD, Krieger JN, Berger RE, Boag AH, Young ID. Consensus development of a histopathological classification system for chronic prostatic inflammation. BJU Int. 2001;87(9):797–805.

Penna G, Mondaini N, Amuchastegui S, Degli Innocenti S, Carini M, Giubilei G, Fibbi B, Colli E, Maggi M, Adorini L. Seminal plasma cytokines and chemokines in prostate inflammation: interleukin 8 as a predictive biomarker in chronic prostatitis/chronic pelvic pain syndrome and benign prostatic hyperplasia. Eur Urol. 2007 Feb;51(2):524-33.

Propert KJ, Alexander RB, Nickel JC, Kusek JW, Litwin MS, Landis JR, et al. Design of a multicenter randomized clinical trial for chronic prostatitis/chronic pelvic pain syndrome. Urology. 2002 Jun 1;59(6):870–6.

Roberts RO, Jacobson DJ, Rhodes T, Klee GG, Leiber MM & Jacobsen SJ. Serum sex hormones and measures of benign prostatic hyperplasia. The Prostate. 2004;61:124-131.

Rohrmann S, Smit E, Giovannucci E, Platz EA. Associations of obesity with lower urinary tract symptoms and noncancer prostate surgery in the Third National Health and Nutrition Examination Survey. Am J Epidemiol. 2004 ;59:390-397.

Schatzl G, Brössner C, Schmid S, Kugler W, Roehrich M, Treu T, Szalay A, Djavan B, Schmidbauer CP, Soeregi S & Madersbacher S. Endocrine status in elderly men with lower urinary tract symptoms: correlation of age, hormonal status, and lower urinary tract function. The Prostate Study Group of the Austrian Society of Urology. Urology. 2000;55:397-402.

Seim A, Hoyo C, Ostbye T, Vatten L. The prevalence correlates of urinary tract symptoms in Norwegian men: the HUNT study. BJU Int. 2005;96:88-92.

Shigehara K, Sugimoto K, Konaka H, Iijima M, Fukushima M, Maeda Y, Mizokami A, Koh E, Origasa H, Iwamoto T, Namiki M. Androgen replacement therapy contributes to improving lower urinary tract symptoms in patients with hypogonadism and benign prostate hypertrophy: a randomised controlled study Aging Male.

2011;14:53-58.

St Sauver JL, Jacobson DJ, McGree ME, Girman CJ, Klee GG, Lieber MM & Jacobsen SJ. Associations between longitudinal changes in serum estrogen, testosterone, and bioavailable testosterone and changes in benign urologic outcomes. Am J Epidemiol. 2011;173:787-796.

Tan AH, Gilling PJ, Kennet KM, Fletcher H & Fraundorfer MR. Long-term results of high-power holmium laser vaporization (ablation) of the prostate. BJU International. 2003;92:707-709.

Tomita K, Mizoue T, Matsumoto T. Lower urinary tract symptoms in relation to lifestyle and medical conditions in Japanese workers. Int J Urol. 2009;16:493-498.

Trifiro MD, Parsons JK, Palazzi-Churas K, Bergstrom J, Lakin C & Barrett-Connor E. Serum sex hormones and the 20-year risk of lower urinary tract symptoms in community-dwelling older men. BJU International. 2010;105:1554-1559.

Vermeulen A, Verdonck L, Kaufman JM. A critical evaluation of simple methods for the estimation of free testosterone in serum. J Clin Endocrinol Metab [Internet]. 1999 Oct [cited 2016 Dec 12];84(10):3666–72.

Vicari E. Diagnostic approach and therapeutic strategy in 133 infertile patients with astheno-necrozoospermia. Arch Ital Urol Androl. 1999 Feb;71(1):19-25.

Vignozzi L, Morelli A, Filippi S, Comeglio P, Chavalmane AK, Marchetta M, Toce M, Yehiely-Cohen R, Vannelli GB, Adorini L, Maggi M. A Farnesoid X receptor activation improves erectile function in animal models of metabolic syndrome and diabetes. J Sex Med. 2011;8:57-77.

Vignozzi L, Morelli A, Sarchielli E, Comeglio P, Filippi S, Cellai I, Maneschi E, Gacci M, Carini M, Piccinni MP, Saad F, Adorini L, Vannelli GB, Maggi M. Testosterone protects from metabolic syndrome-associated prostatitis: an experimental study in rabbit. J Endocrinol. 2012 Jan;212(1):71-84.

Wang C, Nieschlag E, Swerdloff R, Behre HM, Hellstrom WJ, Gooren LJ, et al. Investigation, treatment, and monitoring of late-onset hypogonadism in males: ISA, ISSAM, EAU, EAA, and ASA Recommendations. Eur Urol. 2009;55(1):121–30.
