# Supplementary material for: Testosterone does not affect lower urinary tract symptoms while improving markers of prostatitis in men with benign prostatic hyperplasia: a randomized clinical trial
Source: J Endocrinol Invest. 2022 Mar 17;45(7):1413–25. doi: 10.1007/s40618-022-01776-9 (PMC9184417; doi:10.1007/s40618-022-01776-9)
Supplement: Supplementary file 2 — Supplementary file2 (DOCX 14 KB) [file 40618_2022_1776_MOESM2_ESM.docx]

**Supplementary Table 1. Association of metabolic parameters and medications with the inflammatory score**

| **Factor** | **B (95% confidence interval)** | **p** |
| --- | --- | --- |
| Blood pressure ≥135/85 mmHg or treatment | -0.147 (-0.704; 0.409) | 0.600 |
| Triglycerides ≥150 mg/dL or treatment | -0.262 (-0.870; 0.347) | 0.395 |
| HDL-cholesterol <40 mg/dL or treatment | -0.090 (-0.782; 0.603) | 0.796 |
| Glycemia ≥100 mg/dL or treatment | 0.657 (-0.022; 1.335) | 0.058 |
| Waist circumference ≥102 cm | -0.410 (-1.055; 0.234) | 0.208 |
| Blood pressure ≥135/85 mmHg | 0.509 (-0.413; 10.431) | 0.268 |
| Triglycerides ≥150 mg/dL | 0.381 (-2.141; 2.903) | 0.737 |
| HDL-cholesterol <40 mg/dL | 1.750 (-0.618; 4.118) | 0.127 |
| Glycemia ≥100 mg/dL | -0.333 (-3.452; 2.785) | 0.826 |
| Antihypertensive medications | 0.102 (-0.612; 0.816) | 0.775 |
| Hypolipidemic medications | -0.160 (-0.851; 0.531) | 0.644 |
| Hypoglycemic medications | 1.074 (1.293; 1.854) | 0.008 |

The metabolic parameters were defined according to the National Heart, Lung, and Blood Institute - American Heart Association, using the complete definition of the factors of the metabolic syndrome or separating the clinical/biochemical factors and the medications.
